# Interprofessional Near-Peer Mentoring Teams Enhance Cancer Research Training: Sustainable Approaches for Biomedical Workforce Development of Historically Underrepresented Students

**DOI:** 10.15695/jstem/v5i2.10

**Published:** 2022-08-31

**Authors:** J.J. Huerta, M.T. Figuracion, A. Vazquez-Cortes, R.R. Hanna, A.C. Hernandez, S.B Benitez, M.N. Sipelii, T.C. Brooks, D.T. ZuZero, F.M.R.V. Iopu, C.R. Romero, A. Chavez, A. Zell, S.R. Shugerman, J.S. Shannon, L.K. Marriott

**Affiliations:** 1 Oregon Health and Science University, Portland, OR; 2 Purdue University, West Lafayette, IN; 3 Portland State University, Portland OR; 4 National University of Natural Medicine, Portland, OR

**Keywords:** Science Education, Health Inequities, Disparities, Undergraduate, High School, Professional Development, Program Evaluation, Scholar Research, Cultural Diversity, Interpersonal Relations, Research Education

## Abstract

A cancer research training program explored different approaches for staffing their in-person and virtual programs for high school students. The inclusion of undergraduate near-peer mentors had a universal benefit when implemented across in-person and virtual training programs of one- and ten-week durations. Benefits are described for four stakeholder groups: the high school trainees, program staff, scientist partners, and peer mentors themselves. Peer mentors described that their involvement enhanced their own professional development and, for some, drove a new interest in cancer research. Scientist partners described that peer mentors helped translate their work in the virtual environment for high school students. High school trainees reported their sessions with peer mentors to be one of their favorite parts of the program. Interprofessional peer mentors were highly relatable to students and modeled communication and paths in biomedical research. Staff reported that peer mentors supported student engagement during community shadowing sessions, allowing staff to focus on developing the shadowing experiences with partners. The benefit of including peer mentors was substantial from all viewpoints explored. Their intensive inclusion in cancer research training programs supports sustainability and capacity building in biomedical workforce development.

## INTRODUCTION

Advances in biomedical research depend on innovation, with the increasingly complex nature of biomedical research requiring new ways to tackle scientific problems. Scientists from historically underrepresented backgrounds produce higher rates of scientific innovation yet their careers are more likely to end prematurely (Hinton et al., 2020). In the 2020 census, historically underrepresented racial/ethnic groups comprised 36.2% of the U.S. population (U.S. Census, 2020) yet only 14% of doctoral degree recipients in 2018 (National Center for Science and Engineering Statistics, 2018d) and 22% of faculty positions (National Center for Education Statistics, 2019). Historically underrepresented trainees across biomedical fields report significant barriers to pursuing biomedical research (Marriott et al., 2021), including lack of representation within biomedical research fields (Chang et al., 2014; Clark Blickenstaff, 2005; Harrison and Tanner, 2018; Moss-Racusin et al., 2012).

The National Institutes of Health (NIH) has called for an increased emphasis on diversity and inclusion within biomedical research (National Institutes of Health, 2019) and funded numerous science education and research training initiatives (Valantine and Collins, 2015; Valantine et al., 2016). These NIH programs often emphasize mentored research training, which can have strong benefits for trainees’ professional development (Keller and Lindwall, 2020; Keller et al., 2017). In addition to formal mentorship, non-formal curricular structures can also enhance training experiences of historically underrepresented undergraduates pursuing biomedical research (Marriott et al., 2021). Undergraduate trainees found strong benefit from talking about their paths with other trainees and near-peer mentors in a non-formal setting, which gave trainees reflection time to develop their own professional identity, identify barriers to research participation, and discuss representation and self-care within academia (Marriott et al., 2021). Trainees described self-care as being integrated into their professional development, placed in the context of navigating systemic trauma (e.g. exclusion and abuse in systems; imposter syndrome; generational poverty; health disparities) as well as when balancing work-life responsibilities, a considerable challenge within academic, medical, and research cultures (Marriott et al., 2021). As understanding how to retain diverse students in research training is critical to enhance representation in the biomedical research workforce (Duffus et al., 2014; Estrada et al., 2016; Hinton et al., 2020; Valantine and Collins, 2015; Valantine et al., 2016), our prior findings suggest that training programs should include non-formal discussion time with trainees, ideally with historically underrepresented trainees from NIH-funded diversity initiatives who have walked the biomedical research paths themselves as first generation college students (Marriott et al., 2021).

The Youth Enjoy Science initiative from the National Cancer Institute trains students in cancer research (National Institutes of Health, 2016). While these training programs may know they will include peer mentorship in their programs, the formal involvement and training backgrounds of peer mentors might not be pre-defined. For example, the format of peer mentorship could be embedded within program activities or comprise 1:1 meetings occurring outside program hours (Keller and Lindwall, 2020). Likewise, peer mentor backgrounds could be undergraduates, graduate students, or postdoctoral trainees. While graduate students pursuing cancer research may be envisioned as an obvious choice for peer mentorship given their experience in cancer and sustained involvement in research through the graduate level, our prior work found strong benefits of interprofessional near-peer mentorship when training undergraduates in biomedical research (Marriott et al., 2021), suggesting that inclusion of trainees outside of cancer research may be highly beneficial. Interprofessional education describes students learning with, from, and about each other (Health Professions Accreditors Collaborative, 2019) and has become an increasingly important component of scientific training programs (Averill et al., 2019; Health Professions Accreditors Collaborative, 2019), including in our recent work with undergraduates, as it supported student-led modeling and exploration of different professional degrees (i.e., M.D., R.N., Pharm.D., M.S.E.) and research paths (Ph.D., M.D./Ph.D., M.S.) as well as career options after graduation (Marriott et al., 2021). Cancer teams are becoming increasingly collaborative and interprofessional (Savage et al., 2018; Puts et al, 2018; Salamone et al., 2018), therefore the inclusion of interprofessional peer mentors in cancer research training programs may be particularly impactful.

This manuscript details how the inclusion of peer mentors evolved over time for a statewide-focused cancer research training program for high school students. We describe the backgrounds and roles of peer mentors across training settings (e.g., in-person residential vs. virtual) and program durations ranging from 1–10 weeks. Outcomes were thematically coded from stakeholder feedback and experiences, which were reported by the program’s target audiences, including trainees, scientist partners, program staff, and the peer mentors themselves. These multiple approaches permit triangulation of impact across stakeholder groups and offer new perspectives and avenues for enhancing sustainability of cancer research training programs using interprofessional peer mentors.

## METHODS

### Setting.

**Figure 1.**
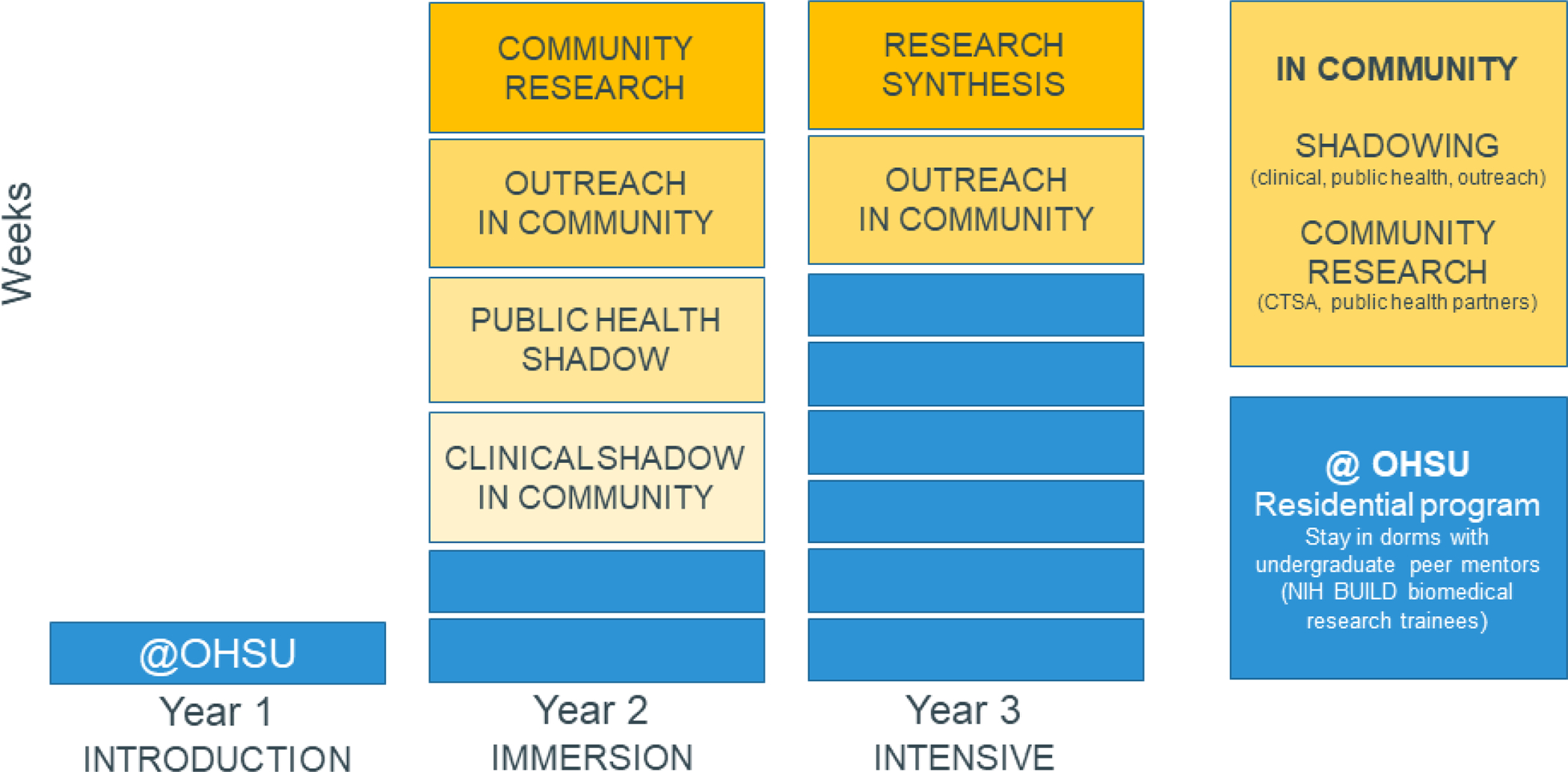
Knight Scholars Program’s three tiers of cancer research training. Blue boxes denote research experiences for Introduction (one week), Immersion (two weeks), and Intensive programs (six weeks), with yellow blocks describing shadowing experiences throughout the state to show additional cancer perspectives.

The Knight Scholars Program (KSP) was developed at Oregon Health and Science University (OHSU; Portland, Oregon), Oregon’s only academic health center and comprehensive cancer center, to train Oregon high school students from historically underrepresented backgrounds in cancer research using approaches that align with the National Cancer Institute’s Youth Enjoy Science initiative (National Institutes of Health, 2016). The program includes a focus on rural students who participate in residential training with urban and frontier students to explore careers in the fields of cancer research, treatment, and prevention (Marriott et al., 2022). Core to the program is its tiered approach across three years that increase in duration and exposure to cancer research (Figure 1), with the first two tiers addressed in this case study. The first program year offers a week-long introduction to cancer researchers while the second year provides immersive shadowing opportunities in clinical care, public health, research, and outreach environments, which showcase the range of research types and careers that are essential to cancer research. The third tier, not yet implemented, includes intensive study in an area of trainees’ choosing. The Knight Scholars Program was reviewed by OHSU’s Institutional Review Board (#18720), with follow-up evaluation of peer mentors overseen by OHSU IRB 22889. The #22889 protocol, entitled “Biomedical Workforce Development” explicitly supports the sharing of training experiences and information to support future workforce development. Peer mentors in this manuscript described their comfort with sharing their experiences and data reported in this manuscript.

### Study Participants.

Peer mentors were recruited from the NIH-funded BUILD EXITO program at Portland State University (PSU) through Enrichment workshops. EXITO is a three-year mentored training program in biomedical research (Richardson et al., 2017). Briefly, sophomore students receive research preparation before being placed in a research learning community for their junior and senior years. EXITO trainees participate in Enrichment workshops, which support contextualization of research training within their professional identity development (Marriott et al., 2021). KSP faculty member (LKM) facilitates Enrichment sessions and has worked with BUILD trainees for five years. An informational flyer and website were shared with PSU EXITO undergraduates. Peer mentors submitted an application (Qualtrics) comprising short essays that were scored on a rubric by two KSP staff (Appendix A). Eligible EXITO trainees at PSU included juniors and seniors for the in-person program, with eligibility expanding to sophomores through seniors for the virtual program. Peer mentor recruitment reach was estimated using course management software (D2L), also used to calculate acceptance rates. In 2021, strongest candidates were interviewed using a script and rubric.

Six peer mentors were hired for the one-week training program in 2019, in which they served as resident advisors and stayed in dormitories with high school trainees (n=25), all of whom were minors under age 18 years. Five peer mentors were hired for the virtual training programs in 2021 during which two programs were run: a one-week Introduction (new high school trainees, n=35) and a ten week Immersion (returning high school trainees; n=15). No program was held in 2020 due to COVID-19 restrictions at the host university. Peer mentors were paid $600 for the 7-day residential program (Introduction) and $5400 for the virtual ten week program (five days per week; Immersion).

### Formative Feedback through Peer Mentor Post-Program Reflections.

Peer mentors provided feedback to program staff (AC and LKM) in one-to-one and group meetings, and via email during and after the program, with notes and lessons learned used for quality improvement throughout the program as well as documentation of peer mentor roles and activities. In 2021, peer mentors of the virtual program shared reflections anonymously using Google Jamboard during the last week of the program to compile reflections and areas for growth for involving peer mentors in cancer research training programs.

### Stakeholder Evaluation.

#### Post-Program Reflection of Peer Mentor Experiences.

After each summer program, peer mentors from that year participated in summative evaluation with the program’s external evaluator (OHSU Evaluation Core; Portland, OR) without the presence of program staff. After the 2019 program, peer mentors were emailed to schedule a short phone interview (15 min) about their experiences with the program. The objective was to learn more about any professional growth they may have experienced as a result of their participation (script in Appendix B). Peer mentors were not asked for specific feedback about the program itself, as this had already been provided through other feedback sessions. For evaluation of 2021, a post-program focus group (1-hr) was conducted with that year’s peer mentors. The change in approach stemmed from positive findings in 2019 yet an incomplete sample size (four of six peer mentors, 66%) and a 1-month delay between program participation and evaluation. Group discussion helps to understand consensus (Flynn et al., 2018) and enabled probing of themes observed in 2019 interviews. The 2019 interview script was adapted for the 2021 focus group by the OHSU Evaluation Core and KSP (LKM; script in Appendix B), with a new question probing about experiences and qualifications of successful peer mentors (e.g. college standing, research experience). Group perspectives were recorded in Webex.

#### External Evaluation of High School Trainees.

External evaluators (OHSU Evaluation Core) facilitated focus groups with program trainees on their last day of the program, with scripts, procedures and full findings described in Marriott and colleagues (2022). The external evaluation team ran eight focus groups with scholars, including six with Introduction scholars (three focus groups per cohort) and two with Immersion scholars, which captured scholar perceptions upon completion of their respective programs. Themes from Introduction and Immersion focus groups that describe peer mentors were summarized for this manuscript by LKM.

#### Scientist Feedback.

Research learning communities (e.g., laboratory principal investigators and primary staff who were involved with hosting two-day placements of Immersion scholars) were sent an online Qualtrics survey immediately after their scholar hosting experience (Marriott et al., 2022). One question asked “Was having a peer mentor attend your lab rotation helpful? Should we keep that again in the future?” with a five-point Likert scale denoting “Definitely don’t keep peer mentors attending lab rotations with scholars=1; Likely don’t keep=2; No preference=3; Likely keep=4; and Definitely keep peer mentors in lab rotations=5). This question and open-ended prompts that described peer mentors were qualitatively coded and with code applications reviewed by study authors.

#### Staff Feedback.

Knight Scholars Program staff (n=7) debriefed the summer 2021 program and used Google Jamboard to share comments and suggestions anonymously. Staff comprised faculty, community liaisons, and program coordinators, all of whom are co-authors on the trainee experience paper (Marriott et al., 2022) and played key roles in conceptualizing the program, developing its schedule, and arranging experiences with community and scientist partners. Submitted responses were transcribed and thematically coded to understand impact and potential roles for peer mentors in training programs.

### Study Reflection Period.

At the conclusion of the summer 2021 program, it was clear to program staff that there were strong benefits for including peer mentors in programmatic activities with scholars. Peer mentors described interest in continuing with the program to design programmatic features for future scholars, consistent with what 2019 peer mentors reported after the program. Peer mentors became more than dormitory chaperones as originally conceived and became integral members of our program team over the study period. Peer mentors’ continued interest in engaging with the program in an advanced capacity is highlighted by their active role in co-designing the program based on data-driven feedback and lived experience. In line with Community-Based Participatory Research (CBPR) principles for advancing equity (Castleden et al., 2010; Sánchez et al., 2021), KSP reached out to all peer mentors from 2019 and 2021 programs about their continued engagement with the program and if they would want to collaborate to synthesize program findings describing impacts of the peer mentor program. Peer mentors could decline, continue with the program as a study participant, or actively engage in the synthesis of findings together. All chose the latter, enabling our program to use equitable CBPR principles to amplify voices of our historically underrepresented scientists who served in peer mentor roles. Collective co-authorship was considered for peer mentors’ significant contributions provided they met authorship criteria outlined by Layton and Stember (2020).

Peer mentors were not paid for their time after the summer program, including follow-up data collection, data analysis, or manuscript preparation activities that occurred in fall and winter. Peer mentors tracked their contributions on an online spreadsheet (Layton and Stembler, 2020), with contributions reflected in author order. Thus, authors of this manuscript were faculty or staff of the Knight Scholars Program as well as peer mentors, who became integral members of the program team over the study period. The researcher characteristics and reflexivity of our peer mentor co-author team are described, in line with Standards for Reporting Qualitative Research (SRQR; O’Brien et al., 2014) and Consolidated criteria for reporting qualitative research (COREQ; Tong et al., 2007).

The IRB-approved protocol (OHSU #22889) entitled ‘Biomedical Workforce Development’ specifically describes conditions that allow all participants (including peer mentors) to engage in data collection and analysis activities, as well as define the sharing permissions for their own information. As the goal of the IRB protocol is to support the training of the future biomedical workforce, these IRB-approved sharing considerations allow our peer mentors to share relevant contextual data and considerations for others to walk that same path. Peer mentor co-authors of this manuscript were able to describe their training paths independently and then worked together to summarize how they wanted their information shared.

### Follow-Up Perspectives and Outcomes Survey.

A short electronic survey (Qualtrics) was emailed to peer mentors in December 2021 with all data blind to peer mentors until data collection was complete. The eight question perspectives survey (Appendix C) asked when peer mentors worked with the program (e.g., 2019 residential in-person or 2021 virtual programs), what their area(s) of interest were when they first applied to work with the program and if their area(s) of interest changed as a result of working with the program (Likert scale: 0=Not at all; 1= A little bit; 2= A lot), with branching logic asking how their interests changed (i.e., in what areas or ways) if they responded yes. Peer mentors were asked if they pursued any cancer-related training, internships, scholarships, or other mentoring activities as a result of participating with the program. They were also asked to reflect on their most significant professional growth as a result of the program, what they would want NIH or other cancer research training programs to know about involving peer mentors in training programs, and their current career trajectory.

Upon completion of the perspectives survey, an end message prompted peer mentors to complete a companion survey (Qualtrics) where they could complete demographic questions (Appendix C). As peer mentors’ interests and paths were generally known to program staff, the separate demographics enabled privacy in responses, with anonymous data only accessible to one co-author (LKM) who coded each demographic variable in a limited data set to maintain privacy. Summary demographic tables were presented to the study team for review of variables to include in the manuscript, all of which were included. Demographic questions included open fields for age, gender, preferred pronouns, and languages spoken other than English. Racial and ethnic groups were measured using REALD (McGee, 2020) and up-coded to NIH inclusion report categories, consistent with measurement approaches for inclusive demographics (Mekinda et al., 2022). Historically underrepresented populations in biomedical sciences were defined using NIH diversity criteria for characterizing underrepresented racial and ethnic groups (National Institutes of Health, 2019), including Blacks or African Americans, Hispanics or Latinos, American Indians or Alaska Natives, and Native Hawaiians or other Pacific Islanders). Also collected were self-reported disability and disadvantaged background, the latter determined by meeting two or more of the seven criteria (National Institutes of Health, 2019), including homelessness experience, foster care experience, Federal Free and Reduced Lunch Program eligibility, first generation college student status, Federal Pell grant eligibility, Special Supplemental Nutrition Program for Women, Infants and Children (WIC) eligibility, and rural geography as defined by self-report for Health Resources and Services Administration (HRSA) Rural Health Grants eligibility (2020) or a Centers for Medicare and Medicaid Services-designated Low-Income and Health Professional Shortage Area (HPSA). HPSA eligibility was verified using peer mentor-reported zip codes against HPSA PY2020 data (CMS, 2020). Addresses were not collected, so HRSA eligibility was not verified. First generation college student status was verified against peer mentors’ self-reported parent/guardians’ educational attainment by LKM.

### Qualitative Data Analysis.

In cases where the OHSU Evaluation Core performed external evaluation, thematic analysis was performed by their faculty and staff using Dedoose and Taguette qualitative coding software, including peer mentor interviews and trainee focus groups. The 2021 peer mentor focus group was facilitated by the OHSU Evaluation Core who de-identified the transcript, with thematic analysis performed using Taguette qualitative software. Focus group codes and excerpts were exported to Excel for secondary analysis by the study team based on a coding dictionary (Appendix B). Columns were used to permit co-coding of themes, as necessary. Follow-up survey data of peer mentor perspectives were exported from Qualtrics into Microsoft Excel to analyze counts and themes. Open-ended prompts were thematically coded by two researchers. Coders met virtually and came to agreement on themes based on a coding dictionary with data definitions (Appendix C). Qualitative data coding occurred in Excel with applications verified by the study team. Coded themes were summarized into matrices for each survey prompt. Qualitative feedback from research learning communities and program staff were compiled, coded, and descriptively analyzed using Microsoft Excel and Word.

## RESULTS

### Study Participants.

A total of 11 peer mentors participated in the cancer research training program, including six in the in-person residential program (1-week duration) and five in the virtual programs (one and ten week durations). Approximately 7% of eligible BUILD EXITO trainees at PSU applied to be a peer mentor, with half (49%) accepted (Table 1). Peer mentors were selected based on an application rubric described in the Appendix, with higher scores reflecting a desire to mentor students and scholars with prior research or lived experience in the community, which would make peer mentors relatable to scholars. As all were in BUILD EXITO, which itself is an interprofessional research training program, we received applicants from across scientific disciplines, including pre-medicine, speech and hearing sciences, public health, and others. Peer mentor backgrounds were not matched to scholar interests, though peer mentors who represented diverse backgrounds and majors were selected, when possible.

#### Peer Mentor Demographics.

Peer mentor demographics were summarized at the time of follow-up (Table 2), with all peer mentors responding characterized as underrepresented in biomedical sciences (11/11; 100%). Peer mentor demographics included all NIH-defined racial and ethnic groups except American Indian/Alaska Native, with 8 of 11 respondents (73%) identifying a historically underrepresented racial/ethnic background (e.g., Black or African American, Native Hawaiian/Pacific Islander, Hispanic or Latino backgrounds represented). All peer mentors (11/11, 100%) reported disadvantaged backgrounds defined by meeting two or more of the seven criteria (National Institutes of Health, 2019). The average number of disadvantaged background criteria met was 4.6 out of 7 (SD=1.1).

Nine of 11 (82%) self-identified as first-generation college student status, which increased to 10 of 11 (91%) when verified against their self-reported parent/guardians’ educational attainment. A discrepancy was observed for Health Professional Shortage Area (HPSA) eligibility when zip codes were verified using PY2020 data (CMS, 2020), with over half (6/10; 60%) incorrectly describing HPSA eligibility. Specifically, 45% of peer mentors (5 of 10) indicated HPSA eligibility, though 70% qualified when their zip codes were verified.

#### Peer Mentor Backgrounds.

All peer mentors were current or former trainees of the NIH-funded BUILD EXITO program, with 3/11 (27%) participating after sophomore year, 4/11 (37%) after their junior year, and 4/11 (37%) after their senior year. Seniors had completed EXITO training but not all seniors had graduated from the university at their time of participation and were completing final graduation requirements at their time of participation. BUILD EXITO scaffolds mentored research training, with sophomores having completed an academic year (three quarters) of research preparation and non-formal peer discussion (termed Enrichment; for review see Marriott et al., 2021). Sophomores had no formal hands-on research training at the time of their peer mentor participation (though they had been matched with their research placements that would start the following fall). Juniors had completed one year of mentored research training and two years of Enrichment, while Seniors completed two years of mentored research training with a research learning community and three years of Enrichment. Average research experience of peer mentors at the time of their participation was 1.1 years (SD=0.8 years). Peer mentors averaged 2.1 years of preparatory experience through EXITO Enrichment (SD=0.8 years). More sophomores (n=3) participated in 2021, comprising 60% of the peer mentor team that year. Average research experience was 1.5 years in 2019 and 0.6 years in 2021, with Enrichment experience averaging 2.5 and 1.6 years, respectively, at the time of peer mentor participation.

### Stakeholder Evaluation.

#### Peer Mentor Post-Program Reflections.

Peer mentors provided feedback toward the end of the program using Google Jamboard that summarized their roles, anticipated impacts, and opportunities for growth (Appendix D). Themes described comfort of trainees with peer mentors, a team approach used by peer mentors to provide mentoring, and an interest in taking more leadership roles in the future. Peer mentors led projects with trainees, including community research projects, photovoice, and informational interviews. They also helped with program activities such as daily evaluations, creating training web pages, and helping to develop mentor awards (e.g., scholars nominated professionals for excellence in communication categories such as mentoring, teaching, research; Marriott et al., 2022). Peer mentor projects also included music playlists that punctuated the day’s sessions, inspired by public health mixtapes of health-equity-related songs (Petteway, 2021). While equity mixtapes weren’t developed, scholar-submitted songs strengthened connection between trainees and peer mentors. Peer mentors cited wanting to spend more time with trainees during their research experiences, which would help them better debrief sessions and provide experiential learning in cancer research.

#### Peer Mentor Post-Program Self-Assessment of Experiences.

After the summer 2019 program, participating peer mentors were contacted by email and asked to participate in a short phone interview. Six peer mentors were contacted and four responded and were subsequently interviewed (67%). All five (100%) participated in 2021 focus groups that probed for themes reported in 2019. Universally positive benefits were reported for including peer mentors in both in-person and virtual training cancer research training programs for high school. (Table 3). Core themes emerged around benefits for career development, skills gained, personal and professional development, mentorship, and diversity (full summaries in Appendix B). Themes from 2019 interviews were used to code 2021 focus group data, with questions added in 2021 to understand considerations around peer mentor qualifications and recommendations for future programs.

In 2019, all reported some level of experience in mentoring prior to being a peer mentor, although not directly with high school students. Peer mentors in both years agreed that their mentoring skills had improved through the program. When probed in 2021, peer mentors emphasized developing their leadership and teamwork skills in particular. Peer mentors described having others in a similar role (i.e., mentoring team) was helpful in making the job better and easier as a mentor. They reported strong benefits of working as an interprofessional mentoring team who could provide different perspectives and expertise. In both years, peer mentors described that sharing similar backgrounds as trainees enhanced their relatability. Overall, the peer mentor experience was impactful for reinvigorating interest and career trajectories in biomedical sciences (Appendix B). Peer mentors reported increased impacts when they were able to co-experience cancer research training experiences with high school students, enabling them to better debrief with students while also supporting their own professional development in cancer research training. Interest in returning to mentor with the program was high.

#### Post-Program External Evaluation of High School Trainees.

Benefits were also reported by high school trainees who described peer mentors as a major theme when asked in focus groups “What parts of the program did you like the most?” (Marriott et al., 2022; summarized excerpts in Appendix B). Trainees liked meeting diverse researchers and professionals who provided wide exposure to cancer careers across work environments and cancer topics. High school trainees emphasized the importance of peer mentors’ relatability (i.e., being from similar backgrounds and close in age), which enabled trainees to feel they had supportive guidance through their cancer research training and experiences. High school trainees in all programs wanted peer mentors to return, regardless of whether the program was offered as a one-week residential or virtual program, or a ten week virtual program.

#### Scientist Feedback.

Due to COVID-19, research rotations and shadowing experiences were virtual. Trainees were matched with four rotations comprising 2-day experiences based on trainee interests. Nine research learning communities participated in summer 2021 and eight unique scientist partners provided feedback (Marriott et al., 2022). Excerpted data relating to peer mentors showed all scientists (100%) reported having a peer mentor in their virtual sessions helpful, with 8/9 (89%) reporting to “definitely keep peer mentors in lab rotations” and 1/9 (11%) indicating to “likely keep” them. The average score was very positive (Average=4.9 out of 5.0; SD=0.3). Labs reported “really appreciat[ing] having the peer mentor present […as they…] often helped with engaging the student scholars.” Recommendations included allowing them to meet the peer mentors earlier so they “can get input from them on what to do.”

#### Staff Feedback.

Staff were universally in favor of peer mentors continuing with the program and observed strong connections between high school trainees and undergraduate peer mentors, finding them helpful for liaising between program staff and students. Staff reported that peer mentors could facilitate and co-experience more research activities with scholars (e.g., research rotations, shadowing, sessions with presenters), which would solve staffing challenges while potentially providing better experiences for trainees (Appendix D). Staff described logistics related human resource classifications and early onboarding, which would support earlier engagement and planning with peer mentors since their interest in ongoing participation was already known.

#### Follow-Up Perspectives and Outcomes of Peer Mentors.

All 11 peer mentors completed the follow-up perspectives survey (100%), representing six from the 2019 in-person program (55%) and five from the 2021 virtual program (45%). Post-program themes (Table 3) were probed in follow-up perspective surveys, with questions asking about interests over time to understand how the experience may have influenced their training paths. Data were coded for each survey question based on prior themes observed. Coded data revealed strong impacts of the program on peer mentors’ professional development (Table 4), with expanded themes and examples described in Appendix C.

Core themes centered around strong growth in professional communication, with the program influencing confidence, comfort, and skills in mentoring others. Peer mentors described that their participation “improve[d their] communication skills, pedagogical knowledge, and cultural competence.” All peer mentors described pursuance of biomedical sciences in their future careers (100%) and all cited mentoring (100%) in their current and future goals (Table 4; Appendix C). Many (64%) cited research in their career trajectories. When asked if their area(s) of interest changed as a result of working with the Knight Scholars Program, 82% said it changed either “a little” (4/11; 36%) or “a lot” (5/11; 45%) with an average change score of 1.3 (SD=0.8). Likewise, 73% of peer mentors described an increased interest in cancer as a result of working with the program, with 5/11 (45%) indicating their interest increased “a little” and 3/11 (27%) indicating “a lot”.

Peer mentors described the experience as “eye-opening” and how it inspired them to continue with mentorship in other areas (e.g., tutoring) and with other training programs supporting high school through graduate students. When asked what they would want NIH or other programs to know about involving peer mentors in research training programs, a prominent theme was their insight and relatability to trainees because they “just went through a similar experience”. Peer mentors described their ability to liaise between students, staff, and scientists in meaningful ways that permitted early identification of emergent issues and opportunities to co-design training programs that improved experiences for target audiences.

## DISCUSSION

Undergraduate peer mentors offer a robust strategy for building workforce capacity in biomedical research. Peer mentors were highly relatable to high school trainees in our program and supported their mentored guidance in cancer research across in-person and virtual contexts of 1–10-week durations. All stakeholder groups reported peer mentors to be highly beneficial, including high school trainees, program staff, scientist partners, and the peer mentors themselves. Peer mentors formed an interprofessional mentoring team to improve research training experiences for underrepresented high school students. Peer mentors described significant growth in their professional communication as a result of the program (82%), which was described in the context of leadership and teamwork. All wanted to continue biomedical sciences (100%) and mentoring (100%), with 73% describing an increased interest in cancer. This case study offers strong support for involving peer mentors in cancer research training programs, which may help to retain diverse students in research training necessary for enhancing representation in the biomedical workforce (Duffus et al., 2014; Estrada et al., 2016; Hinton et al., 2020; Valantine and Collins, 2015; Valantine et al., 2016).

Peer mentors served varied roles across the course of the program, including resident advisors in dormitories during the in-person program, where they chatted with scholars about their day (formal and informal debriefs). In the virtual program, peer mentors led online debrief sessions with scholars in the afternoon that were consistently highly rated by scholars. Likewise, scholars appreciated having peer mentors in longer networking sessions with cancer professionals to facilitate communication. Scholars reported most comfort and interest in talking with near-peer mentors, which occurred in both formal and informal settings modeled after BUILD EXITO Enrichment workshops (Marriott et al., 2021). The strongly positive impacts on trainees and peer mentors alike were observed for both in-person and online settings. These consistent outcomes reported across residential and virtual programs of differing lengths (one week and ten weeks) suggests a strong and important role for peer mentors that can enhance research training programs. Peer mentors represent the backgrounds of our scholars and demonstrated growth in leadership and professional communication, essential skills for scientists. We see strong avenues for sustainability that support diversity goals within the biomedical workforce by building bridges between trainee levels during research training programs.

A major strength of this case study is the documentation that peer mentors with less hands-on research experience can be highly beneficial to research training programs. Peer mentors comprised BUILD trainees in various stages of pursuing independent research. Their diverse paths and interests modeled for high school students what research, academic, and personal paths could look like. Approximately half of our high school trainees were first-generation college students (Marriott et al., 2022) and cited being highly interested in peer mentors’ experiences for getting into college and navigating the STEM environment. As sophomore peer mentors knew their preliminary research paths but had yet to begin their own research training, they were open to shadowing experiences with scholars and helped trainees relate to the new experiences and environments. Having a range of research experience within our peer mentor team was helpful; more research-experienced peer mentors chose to lead scholars in their community research projects. The terms “near-peer mentor” and “interprofessional peer mentor” describe the conditions in which peer mentors interacted with students. Near-peer refers to peer mentors who are close in age or identity. For example, in our study, peer mentors were predominately undergraduates (63%) though 37% were post-baccalaureate. Some of the undergraduate peer mentors were older students returning to school while others were very close in age to high school scholars. The diversity in peer mentor backgrounds, combined with their focus on different scientific disciplines and how those fields can work together interprofessionally, demonstrated the different paths that our scholars could take to pursue cancer research.

Peer mentors were not required to be interested in or studying cancer research at their time of application, yet after the program 73% described an increased interest in cancer, a third (33%) described intentions or pursuance of additional cancer-related training, and one (9%) explicitly cited cancer research as their career trajectory. Even without explicitly describing cancer in their trajectories, peer mentors described interest in fields directly related to cancer, including biomedical informatics, clinical psychology, and clinical care. Peer mentors cited that the interprofessional teams enabled them to recognize their own strengths and those of their team, which was a prominent theme observed in follow-up evaluation. Consistent with our prior work, we found the training program’s interprofessional focus enabled students across physical, biological, clinical, and social sciences to share similar experiences and feelings about professional identity development, which helped others realize they were not alone when questioning how they could become a scientist and what it means to be one, which was “key for underrepresented professionals to distinguish personal barriers from systemic barriers, and identify when solutions needed more individual effort or better access to systems” (Marriott et al., 2021). As described by one Knight Scholars Program peer mentor:

Working with the youth reminded me of how scary it is to be a first-generation and underrepresented high school student trying to figure out whether college or STEM was meant for me. Hearing their stories, lived experiences, and struggles made me realize that we can help students individually but what needs to change is the system and approach. I originally wanted to become a doctor (MD) but this entire experience reminded me of the type of legacy I want to leave behind, which is to help communities and change institutionalized systems to make the process more equitable, experience more endurable, and process/ funding sustainable, which is why I’ll be pursuing a dual medicine/ political role.

The feed forward impact of peer mentor participation was substantial and highlights a team science approach for improving research training programs for youth. Team science supports scientific advancements in biological, physical, and social sciences (Tigges et al., 2019) and is core for translating scientific discoveries (National Center for Advancing Translational Sciences, 2017). Research teams are cited more frequently and have greater scientific impact than individual researchers (Fortunato et al., 2018; Jones et al., 2008; Wuchty et al., 2007; Uzzi et al., 2013; Larivière et al., 2015; Tigges et al., 2019; Hall et al., 2018). This program supported peer mentors in forming a functional team who played an increasingly important role over the course of the cancer research training program. Their involvement increased to attending all research experiences with students and co-creating content with the program team. Shadowing experiences were virtual in 2021 due to COVID-19, which allowed the program to include peer mentors in shadowing experiences without concern for physical space limitations. The positive impacts of including peer mentors were reported across stakeholder groups, including students, staff, and scientists. Peer mentors facilitated session communication and liaised between stakeholders, elevating their participation to leadership roles. They offered important conduits for identifying emergent issues and worked with our program staff to co-create solutions. Using trainees’ daily ratings/feedback, peer mentor feedback, and staff communication, we interrupted three research training experiences hindered by poor communication. Peer mentors alerted program staff after the session, who reached out to labs by email the evening before the second day’s placement and provided suggested changes recommended by the peer mentor. In all cases, scholars’ ratings of the research experience increased the second day; the changes recommended by the peer mentor reflected in scholars’ feedback about the experience as strengths. The labs, who provided feedback about the program in post-experience surveys, cited the peer mentors’ involvement as helpful for bridging communication with scholars and 100% of scientists recommended keeping them in the cancer research training program. Similar approaches could be used to interrupt microaggressions, racism, misogyny, or other harmful practices to inclusive science by guiding programmatic training efforts around cultural competency and helping stakeholders learn how to navigate difficult conversations.

Representation matters for diverse trainees in biomedical research, with 64% of peer mentors describing relatability to trainees as a strong benefit that helped them provide valuable insight, academic support, share recent similar experiences, and offer comfort since they had just walked the same path. All peer mentors of our cancer research training program were historically underrepresented, as defined by NIH criteria (National Institutes of Health, 2019). When asked what NIH or other programs should know about involving peer mentors in training programs, relatability was emphasized as important resource for helping new students understand potentially unknown paths:

Peer mentors afford students an opportunity to navigate and grow in an often unfamiliar pathway. This becomes paramount for underrepresented students in less pursued academic pathways such as STEM. In addition, as a peer-mentor, I have benefited from mentor-student relationships. I have been able to improve my communication skills, pedagogical knowledge, and cultural competence.

As program peer mentors were once mentees in BUILD EXITO, they connected mentor-mentee relationships as important for supporting growth during biomedical training in ways that may be more challenging for program staff:

As a former mentee, I felt intimidated and shy speaking my mind to the people leading the program but I was always open to my peer mentor. She was close to my age and recently went through what I was going through and she always had the right resources for me. She was also a voice for me when I didn’t want to speak up and called me out when I needed it most.

The sentiment was echoed by another peer mentor who highlighted why this connection is so important for trainees:

Peer mentors are absolutely essential to any program that hopes to prepare a generation of professionals for their work. […] Peer mentors allow younger students to forget the pressures of professionalism long enough to ask the most pressing questions.

Peer mentors play important roles on cancer research training teams; their paths illustrate possibilities that contribute to successful trainee experiences. Peer mentors help surface important questions and issues faced by trainees when navigating career paths. The training experience enhanced professional development of both peer mentors and trainees (Marriott et al., 2022), which highlights a sustainable approach for enhancing biomedical workforce development and downstream health equity. About a quarter of peer mentors (27%) cited that they played important liaison roles that helped the program improve experiences for trainees and almost one fifth (18%) explicitly described that peer mentors helped reiterate trainees’ sense of belonging: “*Integrating peer mentors into training programs could reassure [trainees] that they belong in the sciences.*”

Peer mentors were a critical part of the Knight Scholars Program training team who helped translate program activities for trainees, such as shadowing experiences and community research projects. Most peer mentors (82%) cited growth in professional communication as a result of the program, including their “ability to convey thoughts and information to others in a digestible fashion.” Professional communication with trainees, staff, and scientists had transferable benefits on peer mentors’ confidence and networking:

For me, it was improving my networking skills. I had the opportunity to meet so many professionals over the summer, which helped me expand my network. It was also beneficial because talking with professionals helped ease the anxiousness that I experienced when speaking with experts in their fields of work. This growth became noticeably apparent during the program’s final weeks when I had to staff shadowing sessions. I felt confident in my ability to communicate with professionals vocally and via email.

While these strong benefits may suggest to some individuals that peer mentors would volunteer for such positions, we strongly advise programs to offer paid placements. All of our peer mentors are from a self-reported disadvantaged background, with 90% eligible for need-based financial aid through Pell grants. Almost half have experienced house-lessness in their lifetimes (45%). Therefore, offering financial incentives (e.g. $15/hour) to our peer mentors enabled them to focus intently on the scholars, while minimizing barriers to paying rent or affording food. The high cost of living in Portland and many other cities around the country needs to be adequately considered when implementing research training programs. Without compensation, peer mentors who represent higher socioeconomic backgrounds may apply at disproportionately higher rates because they have the luxury to afford a volunteer position. Yet, if we are to engage and retain historically underrepresented trainees, we find strong benefits of including peer mentors from backgrounds that are representative of the trainees. As many of our program’s scholars are also from disadvantaged backgrounds, we find that paying peer mentors for their participation enables individuals from all backgrounds to participate, resulting in strong outcomes that benefitted every stakeholder group measured.

When defining underrepresentation of our peer mentors, we found that rural eligibility was underreported by peer mentors when verified using zip code. More than half the peer mentors (60%) incorrectly listed their HPSA designation, suggesting this data field may warrant verification to prevent underrepresentation of disadvantaged background eligibility. First generation college student status was underreported by 9% of peer mentors when verified against guardians’ educational attainment, consistent with data from high school trainees who also underreported this important eligibility metric for scholarships and programs (Marriott et al., 2022).

Limitations of this case study include a lack of pre-evaluation with peer mentors beyond application essays, which is included in the evaluation of 2022 program peer mentors (work in progress). Thus, while COVID-19 interrupted peer mentor plans to return between 2019 and 2021, peer mentors from both cohorts applied and were selected to the 2022 summer program, forming an interprofessional leadership program in cancer research mentoring that leverages lessons learned from past summer and enhances experiences for students in the Knight Scholars Program.

Together, strong outcomes were observed for including interprofessional, undergraduate peer mentors in cancer research training programs for high school students. Our project offers robust support for two cross-cutting themes in the 2021–2025 NIH Strategic Plan (National Institutes of Health, 2021) including “promoting collaborative science” and “improving minority health and reducing health disparities.” Inclusion of near-peer mentors in research training programs provided important avenues for recruiting and retaining historically underrepresented students in biomedical research. As these trainees represent the diverse communities served by comprehensive cancer centers, the inclusion of peer mentors offers sustainable ways for training our next generation of diverse, innovative scientists while also supporting reach and trust-building with communities. The experiential learning of peer mentors in mentorship around community cancer issues highlights strategies for mitigating health inequities, enhancing relatable mentorship, and expanding professional development. Synergy across NIH training efforts expands career horizons of trainees and enables cancer research training programs to improve when they are studied in collaboration with the underrepresented students they serve.

## Supplementary Material

Appendix A

Appendix B

Appendix C

Appendix D

## Figures and Tables

**Table 1.** Recruitment and selection of peer mentors for the Knight Scholars Program

Program Year	Recruitment Pool*	Peer Mentor Applications (% of pool)	Acceptance Rate (# selected)

2019	115	8 (7%)	60% (6 peer mentors)
2020	107	6 (6%)	0% (no program)^
2021	137	13 (9%)	38% (5 peer mentors)

* The recruitment pool comprised EXITO trainees enrolled at Portland State University that academic year (AY). LKM has worked with Enrichment and EXITO trainees since 2017. Peer mentors were recruited from junior and senior undergraduates in the first two years (n=59 and 56, respectively in AY 2018–2019; n=58 and 49, respectively in AY 2019–2020), which expanded to recruit sophomores through seniors in AY 2020–2021 (n=46 sophomores, 52 juniors, and 39 seniors). Peer mentors had just completed that academic level (e.g., senior year) at their time of participation as a peer mentor. ^The 2020 summer programs were postponed due to COVID-19.

**Table 2.** Peer mentor demographics at the time of follow-up.

Category	Self-Reported Demographics

Age (Average, SD, Range)*	24.6 years (SD=4.3; Range=20–34 years)
Gender	Masculine/man/boy: 4/11 (36%); Feminine/woman/girl: 5/11 (45%); Non-binary/gender-queer 2/11 (18%)
Preferred Pronouns	He/him/his: 4/11 (36%); She/her/hers 5/11 (45%); They/them/theirs: 2/11 (18%)
LGBTQ+	4 of 11 (36%)
Able to speak languages other than English	7 of 10 (70%), including Spanish, American Sign Language, Samoan, Fijian, and Arabic

*Historically Underrepresented in Biomedical Sciences* **	

Overall	10 of 10 (100%)
Underrepresented Racial/ Ethnic Group	8 of 11 (73%)
Disability	3 of 11 (27%)
Disadvantaged background	11 of 11 (100%)

*NIH Disadvantaged Background Categories*	

Homelessness experience	5 of 11 (45%)
Foster care system experience	0 of 11 (0%)
Free and Reduced Lunch eligibility	9 of 9 (100%)
First generation college student	Self-report: 9 of 11 (82%); Verified: 10 of 11 (91%)
Pell grant Eligible	9 of 10 (90%)
SNAP/WIC Eligible	9 of 11 (82%)
Rural (HRSA or HPSA)	9 of 10 (90%)
HRSA Rural Area	6 of 11 (55%)
Health Professional Shortage Area (HPSA)	Self-report: 5 of 10 (50%); Verified: 7 of 10 (70%)

* Age at time of follow-up survey. Gender categories defined using START (2021).

** Historically underrepresented populations in biomedical sciences were determined using NIH definitions (National Institutes of Health, 2019). Health Professional Shortage Area (HPSA) zip codes were verified using PY2020 data (CMS, 2020). Not all questions were answered; percentages reflect the number of peer mentors answering that item.

**Table 3.** Themes reported by peer mentors after the cancer research training programs for high school students.

Focus Area	Themes from 2019 Interviews (in-person program)	Themes from 2021 Focus Groups (virtual program)

**Career development**	Career trajectory reinvigoration; all said the experience caused them to consider their own plans, resulting in either a shift in plans or reaffirmation of existing plans.	Reinvigorated interest in biomedical sciences (prominent theme), Change in area of focus, Desire to work with specific populations, Desire to continue with mentoring or teaching
**Skills gained**	All generally agreed that their mentoring skills had improved though the program.	Leadership (prominent theme), Teamwork (prominent theme), Professionalism (less prominent theme)
**Personal and professional development**	All reported experiencing some degree of personal growth.	Self-Reflection (prominent theme), Connection to others
**Mentorship**	All appreciated the opportunity to connect personally with students	Guiding scholars as they learn research, Supporting trainees as they balance between academics/personal, Offering safe spaces to talk with trainees about challenging times. Advice to future mentors emphasized building trust before trying to mentor.
**Diversity**	Importance of trainees from diverse backgrounds having mentors from diverse backgrounds	Similar backgrounds as trainees; Benefits of an interprofessional peer mentor team; Diverse prior experiences
**Scholar maturity**	Peer mentors were impressed at how thoughtful and articulate students were for their age, particularly around issues of diversity and inclusion.	Not explicitly described in focus groups
**Qualifications and experiences helpful among peer mentors**	Not asked in interviews	College experience with diverse prior experience in research (ranging from research preparation to prior independent research); Similar backgrounds as trainees, Benefits of an interprofessional peer mentor team
**Recommendations for involving peer mentors in cancer research training programs**	Not asked in interviews	More connection with scholars and prior peer mentors before the program; More shared experiences in research; Transparency around structure and opportunities for getting involved. Advice for future programs recommended a mix of group mentoring and 1:1 sessions that match trainees with preferred mentors who become assigned point of contact. Trainee comfort varied considerably across virtual group sizes; including a combination of sizes is ideal.

**Table 4.** Themes reported at follow-up for peer mentors.

Focus Area	Themes Observed
Area of interest at time of application	Clinical interests (8/11; 73%) Non-clinical interests (6/11; 55%) Population interest (4/11; 36%) Cancer interest (1/11; 9%)
Evolution of interests over time	Mentoring for legacy (6/9; 67%) Cancer-related interest change (3/9; 38%) Non-cancer interest change (4/9; 44%)
Pursuance of cancer after participation	Did not pursue cancer (6/9; 67%) Pursued cancer-related training (2/9; 22%) Intends to pursue cancer-related training (1/9; 11%) Cancer-related training impact (Either pursued or intends to pursue; 3/9; 33%)
Pursuance of mentoring after participation	Pursued mentoring activities (7/10; 70%) Intends to pursue mentoring activities (3/10; 30%) Mentoring impact (Pursued or intends to pursue; 10/10; 100%)
Professional growth from participation	Professional Communication (9/11; 82%) Mentorship (4/11; 36%) Networking (2/11; 18%) Leadership (2/11; 18%) Openness to careers (1/11; 9%)
Advice to NIH and other training programs for involving peer mentors	Relatability of peer mentors to trainees (7/11; 64%) Liaison between trainees, program, and partners (3/11; 27%) Reassurance of trainees’ sense of belonging (2/11; 18%)
Career trajectories	Biomedical Sciences (11/11; 100%) Mentoring (8/11; 73%) Research (7/11; 67%) Graduate school (2/11; 18%) Physician (2/11; 18%) Mitigating inequities (3/11; 27%) Cancer (1/11; 9%)

## ABBREVIATIONS

CBPR: Community-Based Participatory Research
HPSA: Health Professional Shortage Area
HRSA: Health Resources and Services Administration
KSP: Knight Scholars Program
NIH: National Institutes of Health
OHSU: Oregon Health and Science University
PSU: Portland State University
SRQR: Standards for Reporting Qualitative Research
WIC: Women, Infants and Children
