# Supplementary material for: Interprofessional Near-Peer Mentoring Teams Enhance Cancer Research Training: Sustainable Approaches for Biomedical Workforce Development of Historically Underrepresented Students
Source: J STEM Outreach. Author manuscript; Available in PMC 2023 May 11. (PMC10174597; doi:10.15695/jstem/v5i2.10)
Supplement: Appendix A [file NIHMS1893019-supplement-Appendix_A.pdf]

Peer Mentors for Workforce Sustainability – Huerta et al.

Appendix A. Peer Mentor Application and Scoring Rubric

Peer Mentor Application

2021 application used as an example.

Knight Scholars Program
Program Dates: Summer 2021
www.ohsu.edu/knightscholars

Thank you for your interest in helping with the Knight Scholars Program! Applications are due by Monday, April 5, 2021 (11:59 pm).

What the student trainees do: The Knight Scholars Program provides a diverse group of high school students from across Oregon with experiences in cancer research. This year's program will be virtual with lab tours and simulations, interactive activities, research projects, and meetings with OHSU scientists who represent a range of cancer-related careers.

What you would do: We are recruiting individuals of all schooling levels (though primarily undergraduates and post-baccs) to serve as peer mentors to interact with high school students. Help them make sense of their research experiences, frame what training could look like in college, and give them guidance/support as they explore new areas.

Dates: We would want to hire you by June 1 so you have time to onboard with the program before students arrive. Students will be with us from June 21-August 27th across two programs:

- Introduction Program (1 week): Monday, July 12 - Friday, July 16, 2021 with 35 students
- Immersion Program (10 weeks): Monday, June 21 - August 27, 2021 with 22 students

Program information at www.ohsu.edu/knightscholars or contact [program coordinator]

Overview: Mentors are undergraduate students, post-baccalaureate students, graduate students, post-doctoral fellows or research staff who provide support to students while serving as role models during the programs. Mentors offer insight into the life and responsibilities associated with careers in biomedical research, such as cancer. They can have a profound and tangible impact on scholars' educational, career, and life choices through personal discussions about career and educational goals. They can offer advice, support, and insights based on their own experiences. Mentors help scholars understand the benefits, expectations, and environment of a research lab, graduate school, and pre-health professions. In addition, they can also help address concerns that scholars may have about the program, activities, pursuing a degree, academic career, and/or health science career. In these ways and others, mentors help to encourage students' pursuit of a career in the cancer field.

- Be responsible for adhering to policies impacting minors
- Serve as positive role model to scholars
- Provide mentoring
- Provide support with any issues or concerns that may arise
- Assist with session and activities preparation and assignments
- Assist students with preparation and planning for program activities to include Q&A sessions, research projects
- Co-develop web-based materials for sharing information about cancer research with Oregon high schools
- Develop a sense of community among scholars
- Adhere to and enforce OHSU policies and procedures and Code of Conduct
- Serve as liaison between Scholars and Knight Scholars Program staff

Pay scale/Incentives:

- $15.00/hour
- Opportunities to publish and develop educational materials and scientific results
- Add peer mentor role for this NIH-funded program to your CV!

Commitment: June 21-August 27, 2021. We offer two student programs this summer. Flexible schedule from 10-40 hours/week.

Qualifications/Experience: OHSU background check clearance for all selected candidates

- Required: Sophomore through graduate student; Bachelor's Degree/No experience
- Preferred: First generation college student and/or community college transfer student
- Preferred:Participation in STEM-focused researching training (e.g., BUILD EXITO, McNair, LSAMP, etc.) to guide new students. Prior experience mentoring or working with middle or high school students (i.e., classroom, after-school or camp). Experience with the development of website content, infographics, podcasts, or other items for sharing science information

About You

I am applying to be a peer mentor for the following session(s): (Please check all that apply.) It's our hope that you'll work with us all summer, but if you're limited, you can indicate specific weeks.

- Full Summer (June 1-Aug 31): hours can be flexible, but you want to work with students in both programs throughout the summer)
- Introduction Program (1 week): Monday, July 12 - Friday, July 16, 2020 with 35 students
- Immersion Program (10 weeks): Monday, July 21 - August 27, 2021 with 22 students with varied schedule. Scholar/peer mentor sessions are planned for the afternoons (Monday - Thursday) and on Fridays
- Community Research week: Monday July 19 - Friday July 23, 2021 - Scholars will be diving more deeply into researching issues affecting their communities. We'll need some help with student support during the process

First Name [open field]
Last Name [open field]
Email address that you check frequently [open field]
Mobile Phone Number [open field]
Alternate phone number [open field]

School attending:

- PSU
- OHSU
- Other [open field]

Current educational program:

- Undergraduate
- Post-Baccalaureate
- Masters student
- Doctoral student
- Post Doctoral student

Graduation year (expected is fine): [open field]
Major/Degree/Area of study: [open field]
What is your gender? [open field]
What pronouns should we use when contacting you? (e.g., he/him, she/her, they/them): [open field]

Application

Will you be participating in another OHSU summer program?
(Note: There is a 40-hours/week total maximum schedule allowed which may restrict your ability to work with Knight Scholars. We can work with you to figure out if time conflicts apply.)

- Yes
- Maybe
- No

If Yes or Maybe, please specify: [open field]

Do you have any other limitations in hours that we should know about in advance (e.g., EXITO induction or immersion, etc.). We can plan around these activities. [open field]

Short Essay 1: Why do you want to be a peer mentor with the Knight Scholars Program?

Short Essay 2: Please describe any prior experience serving as a research assistant, mentor, resident advisor, camp counselor, or youth mentor (including approximate dates if you know them). It is very important to us that these high school

students have caring and responsible mentors – particularly since they are under 18. Please let us know anything that describes why you would be the right fit.

Short Essay 3: Please describe the qualities you believe a student's parent or guardian would want to see in a mentor. How would you meet their expectations?

Diversity and Representation Short Answer
Knight Scholars are high school students who will be looking to you for guidance. What do you want us to know that will better help us match you with students?

For example:

- **Location**. Students will come from [listed regions in Oregon]. If you grew up in one of these areas, we want to match you with students from that region!
- **Diversity**. We have recruited students who meet NIH's definition of being underrepresented in science, including students from racial/ethnic minorities, students with disabilities, and students who come from disadvantaged backgrounds (e.g., "such as those from low-income families or individuals who come from a social, cultural, or educational environment such as rural or inner-city environments which demonstrably and recently directly inhibited the individual from obtaining the knowledge, skills, and abilities necessary to develop and participate in a research career."). Individuals who identify with these backgrounds are highly welcomed!
- **First generation college student**. Were you the first in your family to attend a 4 year college?
- **Professional struggles.** If you struggled in high school or weren't accepted into a college or graduate degree program right away, your perspective is especially valuable. Science is hard. The goal of this program is for students to envision themselves in your shoes and understand what different paths can look like -- especially when the path doesn't exactly go as planned. Perseverance is gold.
- **Experience with cancer.** You could be working with a cancer researcher, shadowed with a clinician treating cancer, have a friend or family member with cancer, or volunteered with a cancer outreach program. You don't need to have personal or research experience with cancer, but it can be helpful in some cases.
- **Fluency in Spanish**. We have several students who speak Spanish at home. If you are fluent a language other than English, please let us know.
- Also, you don't need to respond to each of these bullets -- they're simply some ways to connect more meaningfully with students.

Optional: Do you have any experience with the development of website content, infographics, podcasts, or other items for sharing science information? It's OK if you don't. Please indicate if you have interest in learning any of these skills,

Demographics for NIH Reporting
The Knight Scholars Program is funded by the National Institutes of Health. They ask that we describe the demographic of our students and those who work with our program. The following questions are used to quantify underrepresented individuals in biomedical science.

Were you or are you currently homeless, as defined by the McKinney-Vento Homeless Assistance Act? Definition: https://nche.ed.gov/mckinney-vento

- Yes
- No
- [Note: Prefer not to answer is recommended for future programs to include]

Were you or are you currently in the foster care system, as defined by the Administration for Children and Families? Definition: https://www.acf.hhs.gov/cb/focus-areas/foster-care

- Yes
- No
- [Note: Prefer not to answer is recommended for future programs to include]

Were you or are you currently eligible for the Federal Free and Reduced Lunch Program or two or more years? Definition: https://www.fns.usda.gov/school-meals/income-eligibility-guidelines

- Yes
- No
- [Note: Don't Know and Prefer not to answer are recommended for future programs to include]

Have/had you parents or legal guardians who did not complete a bachelor's degree? Definition: https://nces.ed.gov/pubs2018009.pdf

- Yes
- No
- [Note: Don't Know and Prefer not to answer are recommended for future programs to include]

Were you or are you currently eligible for Federal Pell grants? Definition: https://www2.ed.gov/programs/fpg/eligibity.html

- Yes
- No
- [Note: Don't Know and Prefer not to answer are recommended for future programs to include]

Have you received support from the Special Supplemental Nutrition Program for Women, Infants and Children (WIC) as a parent or child? Definition: www.fns.usda.gov/wic/wic-eligibility-requirements

- Yes
- No
- [Note: Don't Know and Prefer not to answer are recommended for future programs to include]

Have you grown up in a U.S. rural area, as designated by the Health Resources and Services Administration (HRSA) Rural Health Grants Eligibility Analyzer? Definition: https://data.hrsa.gov/tools/rural-health

- Yes
- No
- [Note: Don't Know and Prefer not to answer are recommended for future programs to include]

What is your race/ethnicity? (Please check all that apply.)

- Black or African American
- Hispanic or Latino
- American Indian or Alaska Natives
- Native Hawaiian or Pacific Islander
- White or Caucasian
- Unknown
- Prefer not to answer
- [Note: REALD (McGee, 2020) is recommended for future programs to include, though if shorter lists are preferred, REALD also includes Middle Eastern/North African in roll-up categories.]

Do you have a disability? Individuals with disabilities are defined as those with a physical or mental impairment that substantially limits one or more major life activities, as described in the Americans with Disabilities Act of 1990, as amended. https://www.nsf.gov/statistics/2017/nsf17310/static/data/tab7-5.pdf

- Yes
- No
- [Note: Don't Know and Prefer not to answer are recommended for future programs to include]

Will you need any disability accommodations while you're with us?

- Yes
- No

    If yes, please describe. [open field]

Terms and Conditions

If you are selected as a finalist, we will:

1. call you for an interview by April 15.
2. ask you for a professional reference (e.g., from an employer, etc.) We don't need this now, but please be aware that we will ask you for it.

- I agree to these terms
- I would like to talk to someone about these terms

Selected candidates will be onboarded through OHSU human resources to include a background check and virtual policy and code of conduct training sessions.

- I agree to these terms
- I would like to talk to someone about these terms

Survey expectations: The Knight Scholars Program is an NIH-funded research study. As such, we will ask you to complete a short evaluation to help us understand your experiences with students and what issues came up. This will likely be by interview or a short survey -- essentially, we want to understand what additional training/support is needed for mentors when working with students.

- I agree to these terms
- I would like to talk to someone about these terms

Terms and conditions: I agree to all the terms and conditions of the OHSU Code of Conduct (http://ohsu.ellucid.com/documents/view/13349/?security=27eb3b339b737a82cd48b3282cdd3be05e75d10e

- I agree to these terms
- I would like to talk to someone about these terms

I have completed this application honestly and to the best of my abilities (please type your name below to sign) [Signature line provided as an open field]

Questions/Comments: (optional) [open field]

Peer Mentor Application Scoring Rubric for 2021 Programs

Instructions: Each application will be reviewed by two people who will score it using the criteria below. Scores will be tabulated on a companion Excel sheet.

Summary of Scoring Components

Section	Component	Points Possible	% of Total Score
A	Short Answer 1: Why do you want to be a Peer Mentor with the Knight Scholars Program?	20	25%
B	Short Answer 2: Please describe any prior experience serving as a research assistant, mentor, resident advisor, camp counselor, or youth mentor (including approximate dates if you know them). It is very important to us that these high school students have caring and responsible mentors– particularly since they are under 18. Please let us know anything that describes why you would be the right fit.	20	25%
C	Short Answer 3: Please describe the qualities you believe a student's parent or guardian would want to see in a mentor. How would you meet their expectations?	15	19%
D	Time availability/conflicts	10	13%
E	Representation	15	19%
F	Recommendation for interview?		
	Total Points	80	100%

Details of Scoring Components and Rubric:

A) Short Answer 1: Why do you want to be a Peer Mentor with the Knight Scholars Program?

Criteria - Reason to participate	Score
Essay not included or completed or non-responsive to question.	0
Unclear, vague description of reason to participate	2
Clear but brief description of reason to participate	6
Compelling description of reason to participate	10

Criteria – What they are hoping to gain	Score
No explanation of what they are hoping to gain	0
Unclear, vague description of what they are hoping to gain	2
Clear but brief description of what they are hoping to gain	6
Compelling description of what they are hoping to gain from KSP	10

B) Total Points (0-20; partial component scores allowed, e.g. 8):__________

B) Short Answer 2: Please describe any prior experience serving as a research assistant, mentor, resident advisor, camp counselor, or youth mentor (including approximate dates if you know them). It is very important to us that these high school students have caring and responsible mentors – particularly since they are under 18. Please let us know anything that describes why you would be the right fit.

Criteria – Mentor Experience	Score
Unclear, vague description of experience.	0
Clear description of experience.	5
Compelling description of experience.	10

Criteria – Connection between experience and interest in being peer mentor in this program	Score
No to minimal connection between experience and interest in this program	0
Clear connection between experience and interest in this program	5
Compelling connection between experience and interest in this program	10

B) Total Points (0-20; partial scores allowed):__________

C) Short Answer 3: Please describe the qualities you believe a student's parent or guardian would want to see in a mentor. How would you meet their expectations?

Criteria – Qualities of mentor	Score
No description of qualities	0
Unclear, vague description of qualities that would be important	1
Some description of qualities	5
Clear description of qualities	10
Compelling description of important qualities	15

C) Total Points (0-15; partial scores allowed):__________

D) Availability: Many EXITO scholars who are still in the program have other requirements in the summer. To what extent do you see a conflict that can't be managed?

Criteria – Availability	Score
No description of availability	0
Serious conflicts exist, unclear how to manage	1
Small conflicts exist, unclear how to manage	5
Conflicts exist (varying extent), but can be managed	6-9
No conflicts; scholar is fully available	10

D) Total Points (0-10; partial scores allowed):__________

E) Potential Fit - Demographics (Experience with Virtual and Creating Content added for 2021)
Knight Scholars are high school students who will be looking to you for guidance. What do you want us to know that will better help us match you with students?

For example:

- **Location**. Students will come from (5 sites named in Oregon). If you grew up in one of these areas, we want to match you with students from that region!
- **Diversity**. We have recruited students who meet NIH's definition of being underrepresented in science, including students from racial/ethnic minorities, students with disabilities, and students who come from disadvantaged backgrounds (e.g., "such as those from low-income families or individuals who come from a social, cultural, or educational environment such as rural or inner-city environments which demonstrably and recently directly inhibited the individual from obtaining the knowledge, skills, and abilities necessary to develop and participate in a research career."). Diversity is highly welcomed!
- **First generation college student.** Were you the first in your family to attend a 4 year college?
- **Professional struggles**. If you struggled in high school or weren't accepted into a college or graduate degree program right away, your perspective is especially valuable. Science is hard. The goal of this program is for students to envision themselves in your shoes and understand what different paths can look like -- especially when the path doesn't exactly go as planned. Perseverance is gold.
- **Experience with cancer**. You could be working with a cancer researcher, shadowed with a clinician treating cancer, have a friend or family member with cancer, or volunteered with a cancer outreach program. You don't need to have personal or research experience with cancer, but it can be helpful in some cases.
- **Fluency in Spanish.** We have several students who speak Spanish at home. If you are fluent a language other than English, please let us know.

Also, you don't need to respond to each of these bullets -- they're simply some ways to connect more meaningfully with students.

Criteria – Scholar background and demographics	Score
No description of demographics	0
Brief description of demographics and/or unclear if applicant's demographics and/or background matches our students	1
Clear description of background; could be a good fit for our students	3
Compelling description of scholar background and would be a good fit for our students	5

Added for 2021 program: Applicants were asked about their experience with virtual education and/or if they had any prior experience creating digital content that could be used in the virtual space.

Criteria – Experience describing virtual	Score
No description of virtual learning/advice	0
Brief description of virtual	1
Clear description of virtual	3
Compelling description of virtual	5

Criteria – Experience describing creating content	Score
No description of creating content (web, podcasts, infographics, etc.)	0
Brief description of creating content	1
Clear description of creating content	3
Compelling description of creating content	5

E) Total Points (0-15; partial scores allowed):_________

F) Should we invite for an interview?

Criteria – Invite for interview	Score
Applicant is not competitive for position	No interview
Applicant is competitive, just not the most competitive. Interview if time/space allows	Maybe
Applicant is highly competitive; definitely interview	Yes

G) Comments

REQUIRED! Please write a brief blurb about the candidate and what you think their strengths/weaknesses are. Please also include a description of student's diversity (e.g., first generation college student, racial/ ethnic background, disadvantaged background, etc.) **Final decisions will use these comments to help decide!**

Peer Mentor Interview Script and Scoring Rubric

Knight Scholars Program – Peer Mentor Interview Questions

**Applicant Name:**			
**Interview date:**		**Time:**	
**Interviewer(s):**		□ Virtual □ Tele □ In person	

§ Provide introductions and brief overview.

§ Do not ask questions related to race/color/national origin, religion, sex/gender identity/sexual orientation, disability, pregnancy status, age, genetic information, citizenship, marital status, or number of children.

§ Rating: 1-Below Expectations, 2-Meets Expectations, 3-Exceeds Expectations

**Questions for all applicants:**	**Interviewer Notes**	**Rating**
1. What motivated you to apply for this opportunity?<br>2. What is your availability June-August 9a-5p? How many hours/week do you think you may realistically be able to give to the project? How will you balance your schedule?<br>3. Do you have reliable technology and internet access this summer?<br>4. What do you think will be the most challenging aspect about working with our KSP students?<br>5. How would you handle a negative and/or disengaged student?<br>6. How would you handle questions surrounding race/ethnic diversity, equity and health disparities that may arise?<br>7. *Students must complete a research project about local cancer issues to help them see the relevance of cancer research and patient care in their own communities.* How comfortable would you feel helping students with their community research project? What would your strategies be for helping students with their community research project?<br>8. *Interviewer rating of communication skills – How well does applicant express themselves?*		
	Total:	/ 24

**What questions do you have for us about your involvement with the program?**<br>*(list their questions)*		

Overall, the candidate is well qualified for the position.

□ Agree □ Somewhat agree □ Neutral □ Somewhat disagree □ Disagree

Recommend for hire: □ Yes □ No

Interviewer notes:
