# Supplementary material for: Interprofessional Near-Peer Mentoring Teams Enhance Cancer Research Training: Sustainable Approaches for Biomedical Workforce Development of Historically Underrepresented Students
Source: J STEM Outreach. Author manuscript; Available in PMC 2023 May 11. (PMC10174597; doi:10.15695/jstem/v5i2.10)
Supplement: Appendix B [file NIHMS1893019-supplement-Appendix_B.pdf]

Peer Mentors for Workforce Sustainability – Huerta et al.
Appendix B. External Evaluation of Peer Mentors and High School Trainees

Scripts
Interviews and focus groups were facilitated by the program's external evaluator (OHSU Evaluation Core).

Table B1. Scripts for post-program external evaluation of peer mentors using interviews and focus groups.

2019 Phone Interview Script (In-person program)	2021 Focus Group Questions (Virtual programs)
• How did being a peer mentor impact you?<br>• Did your participation make you think about your own career trajectory? If so, how?<br>• What did you learn about yourselves through the process of being a peer mentor this summer?<br>• What advice would you have for improving the peer mentor experience?	• How did being a peer mentor impact you? (Prompt: what were some of the things that stood out?)<br>• Did it make you think about your own biomedical research trajectories? If so, how?<br>• What about your interest in cancer research specifically?<br>• What did you learn about yourselves through the process of being a peer mentor this summer?<br>• You are all at different phases in your own training (after sophomore year vs. graduated). How does level (e.g., soph, jr, graduate) impact your ability to be a peer mentor with scholars?<br>• Is this something program organizers should be mindful of in the future? (e.g., is there an ideal time for involving peer mentors or is a range in age/grade/level important?)

2019 Interviews

Interviews and focus groups were facilitated by the program's external evaluator (OHSU Evaluation Core) with their summative report below.

Knight Scholars Resident Advisor Interviews: Program Year 1, 2019
INTRODUCTION

Four peer mentors (PMs) were interviewed about their experiences with the program. The objective was to learn more about any personal growth they may have experienced as a result of participation. The PMs were not asked for specific feedback on the program itself, as this had already been provided through numerous feedback sessions.

METHODS

Participating PMs were contacted by email and asked to participate in a short phone interview. Six PMs were contacted and four responded and were subsequently interviewed. Interviews took place approximately 1 month post-PM experience. Each interview lasted approximately 15 minutes. Interviews were loaded into Taguette, a qualitative analysis software package, and six codes were developed (see Table B2).

Table B2. Themes reported by peer mentors participating in the 2019 in-person program.

Theme	Description	Example Quote
**Mentoring**	Personal experience with mentoring or comment on mentoring	"This mentoring position was the more 'formal appointment' type of position. In the past it was always voluntary."
**Career**	Comment on own career trajectory	"It helped to reflect on why I have done it and it has motivated me - now really interested in public health."
**Diversity**	Issues of diversity	"There are too many diversification programs that are trying to take diverse students and force them to be more white – this doesn't help anyone."
**Scholar maturity**	Comments on overall maturity of Knight Scholars	"We need students of color willing and able to talk about these things. They are only 16 and articulating these points."
**Skills gained**	Specific skills gained	"I got out of it - building skills to work with a younger population than college students."
**Other benefits**	Other personal or professional benefits from participating	"I got to see how privileged I was in terms of me making it this far in my journey and education."

SUMMARY

All four of the PMs interviewed reported experiencing some degree of personal growth. All of them had some level of experience in mentoring prior to being a PM, although not directly with students this age (high schoolers). However, they also generally agreed that their mentoring skills had improved through this work. They appreciated the opportunity to connect personally with the students ("A lot of times you hear stuff from students that are emotional, you need to be professional and be genuine the same time"), and believed that it was important that the scholars who were from diverse backgrounds had mentors from diverse backgrounds. Although they did not necessarily feel that they needed more training as mentors, they did express the feeling that researcher-mentors (in general and those connected to the Knight Scholars Program) needed additional training in working with students from diverse and disadvantaged backgrounds ("I was glad I was in the position I was, I could be a source for these students. Is that guy racist because he is a PhD and he is supposed to be mentoring us? Mentors need more education.")

The PMs were asked specifically if their participation had prompted further thoughts about their own career trajectories. All of them agreed that the experience had caused them to consider their own plans, resulting in either a shift in plans ("Hearing the stories from the cancer doctors their research, kind of shifted me to think about being a Dr. researcher.") or a reaffirmation of existing plans ("It was so rewarding and I learned that I can totally do this. It just kind of clicked.").

Other important themes included comments on the overall maturity of the scholars (PMs were impressed at how thoughtful and articulate they were for their age, particularly around issues of diversity and inclusion, "They are so intelligent, like little adults"), and comments on other benefits of participation as PMs such as learning about flexibility and adaptation in working with youth.

All PMs would like to participate next year if they are available and if the opportunity is available.

2021 Focus Groups

Virtual programs with students of one week and ten week durations.

Summary of themes from the virtual programs

Table B3. Summary of themes from peer mentor focus group in 2021 (virtual programs) describing impact of the program.

Focus area	Themes observed
**Impact on career development**	Reinvigorated interest in biomedical sciences (prominent theme), Change in area of focus, Desire to work with specific populations, Desire to continue with mentoring or teaching
**Impact on skills gained**	Leadership (prominent theme), Teamwork (prominent theme), Professionalism (less prominent theme)
**Impact on personal and professional development**	Self-Reflection (prominent theme), Connection to others
**Mentorship roles**	Guiding scholars in learning research, Supporting trainees as they balance between academics/personal, Offering safe spaces to talk with trainees about challenging times. Advice to future mentors emphasized building trust before trying to mentor.
**Qualifications and experiences helpful among peer mentors**	College experience with diverse prior experience in research (ranging from research preparation to prior independent research); Similar backgrounds as trainees, Benefits of an interprofessional peer mentor team
**Recommendations for involving peer mentors in cancer research training programs**	More connection with scholars and prior mentors; More shared experiences in research; Transparency around structure and opportunities for getting involved; Advice for future programs recommended including a mix of group mentoring and 1:1 sessions that match trainees with preferred mentors that are assigned as a point of contact. Peer mentors also recognized that trainee comfort varied considerably across group sizes and that having a combination of group sizes is ideal.

Impact on career development

Table B4. Impact of Knight Scholars Program on peer mentors' career development.

Code	Definition	Example Quotes
**Reinvigorated interest in biomedical sciences** (prominent theme)	Describes a spark in a new area or renewed interest in science, often described in the context of reaffirming an existing interest in biomedical sciences.	• [The program gave] "a new spark towards research and getting excited about it again. I guess during the research [rotation] weeks, being exposed to the different types of projects [offered a] shift in interest again [and] exposure to other types of research besides which [we] had been working on with [our] own research [in BUILD]."<br>• "I've always known that I've wanted to have some hand in research. Going to the research rotations really re-ignited that fire because doing my research for my research program really burned me out, but having exposure to the other fields of research really [made me] excited to apply it to graduate school. So, to say, it really helped me find my passion again in research doing this program."<br>• Guiding [students] through their community research projects, we saw how passionate they were about it and all the work that they were doing for it, and that kind of also made me excited to pursue research, so your exposure that you got from participating and chatting with the students and also their fresh look on things also kind of contributed to how you were thinking about your own career.<br>• "I think my career path is still the same. This just reinforced it."<br>• "I was already considering a Ph.D. and then we actually had a rotation [in the field of interest] and we had to shadow them virtually, and I was like. 'Yeah, I think I made the right decision' after talking to them and asking them question and I connected with them."
**Change in area of focus**	Describes how the program changed the peer mentors' area of focus in their career trajectory.	• [Before the program] "I didn't really know much about cancer research honestly, so I didn't have any interest in it. But after hearing all the different researchers that we've met and the counselors and partnership panel as well hearing people that survive from cancer, their experiences, resources they had, just made me more interested in public health."<br>• "For me, just seeing those different labs, and how they look at cell cultures and how they work through their labs really made me realize how much I want to work in a lab setting like that because before that, I didn't really see myself in biomedical. But after getting that exposure, it sounds like maybe that's something I want to do in the future. I've always been focused on becoming an M.D clinician because I love interacting with patients, but now I'm realizing that I also love working in the lab and that I'd be interested in conducting research, like, going through cell cultures."<br>• "This experience has made me more interested in public health."
**Desire to work with specific populations**	Interested in working with specific populations, including children, cancer survivors, and communities.	• "For me, it impacted me a lot. It allowed me to actually realize that I actually want to work with kids for my future career."<br>• "Working with the survivorship programs, I find a lot more fascinating than I thought I would.
**Desire to continue with mentoring or teaching**	Peer mentors describe how the program grew interest in continuing with mentoring and teaching in the future.	• "I really love being a mentor and I really hope I can do this in the future. Like, not just [... with] this program [but] with other programs as well"<br>• "Hopefully, if everything goes right I'm still going to be a mentor next year as well."<br>• "This experience has made me change my focus from just pure research and clinical work to going towards research and teaching. I really like the aspect of being a peer mentor and working with the students."

Impact on peer mentors' skills gained

Table B5. Impact of Knight Scholars Program on peer mentors' skills.

Code	Definition	Example Quotes
**Leadership**<br>(prominent theme)	Describes how the program allowed peer mentors to build or flex their leadership skills, including the design of program components in the virtual space to best support trainees (e.g., taking charge of a project and shaping the direction it could go)	• [The program] "allowed me to grow my leadership skills."<br>• "Being a peer mentor this summer has really helped me bring out a lot of my leadership skills"<br>• "I'd like to second what [identifier redacted] said with leadership skills, I've really gotten to reinforce that."<br>• "Since the project [being virtual was] so new, a lot of things weren't organized and planned out. [KSP program staff] let us basically make our own schedule and when we took lead on certain projects, it was really up to us how we wanted to do that, when we wanted to make presentations, [...] and since we were kind of the main point of contact with scholars, we were always someone who initiated our voice, concerns, or listed comments from the scholars, things like that."<br>• "Taking charge of a project and shaping how you think it should be and the direction you want to go is something that I think we all learned with our various projects."
**Teamwork**<br>(prominent theme)	Describes teamwork and collaboration, often in the context of peer mentors recognizing their own strengths as well as those of the peer mentor team.	• "For me, I've had mentoring experience before, but this was still different because [in the prior mentoring experience] it was just me with a group of students and I was the only mentor. So, here, having peer mentors colleagues, I feel like it was much better because if I couldn't cover something, I still had my peers to rely on and I think it was more effective that way, because it was more engaging and everyone had different questions. Like, all the peer mentors would come up with different questions to ask the scholars and so I really enjoyed it. I just felt it was very engaging."<br>• "I really, really love working with the other mentors. That's been one of the biggest highlights of this whole program."<br>• [I learned that] "it's okay to not know everything or to know the answer to every question. As my role as a peer mentor, I always thought 'oh, if a student asks the question, I need to give them a straight answer', but I think I've learned to rely on my peer mentors and say that, 'I'm not sure if I know the answer' to a question and just look it up afterwards and then let them know about it or ask the staff members or peer mentors for help."<br>• "I've learned a lot of collaboration. And also that I learned that I need to learn how to keep focus; it really helps having a lot of different personalities in this program and collaborating because everyone brings different skills and teaching academic skills is not my strong point."<br>• "I feel a lot of pressure doing things myself because I had the tendency to volunteer to do more work, [so] it's nice to have help and [gain] confidence that we can do the project and facilitate things."
**Professionalism**<br>(less prominent theme)	Peer mentors describe how the program helped to develop new skills or reinforce existing skills that can be used in their careers, often described as a set of experiences, such as networking, data use, building CVs, etc.	• "I can go so many ways. More than I thought from when first applied to this position. It's not what I had in mind and it was in a really good way. I'd like to second what [identifier redacted] said with leadership skills, I've really gotten to reinforce that. I really love being a mentor and I really hope I can do this in the future. Like, not just in this program but with other programs as well. I just really love getting to talk to scholars, getting to mentor, getting to become immersed in this program alongside with them. And it's also helped me develop professional skills as well because I have already learned networking with professionals, building my CV, stuff like that. But teaching others to do it just helped reinforce the importance of that and I don't know if I can put it all into words. It's just I really love this experience."<br>• "There were moments of awkward silence where the students would not have any questions and so we were able to build on our skills [a]t the moment of just thinking up a question and ask to fill in that silence, so I feel like that helped us get in our mind and just act."

Impact on peer mentors' personal and professional development

Table B6. Impact of Knight Scholars Program on peer mentors' personal and professional development.

Code	Definition	Example Quotes
**Self-Reflection** (prominent theme)	Peer mentors describe self-reflection about interests, strengths, or areas for future growth	• "Being a peer mentor this summer has really helped me [be more] vocal about the skills that I want to develop and [identifier redacted] was very receptive of what I had to say and provided me opportunities where I can actually develop those skills. And also, I learned I really like being a mentor and I'm not half bad at it. I thought that I would have very low patience for high schoolers, but, you know, I really, really enjoyed the experience."<br>• "It's highlighted things I need to fix, or at least work on."
**Connection to others**	Closer connections to students, staff, and researchers as a result of participating with the program	• **Self-reflection around connection:** "I learned I have better social skills than I thought I did. I didn't know I could relate to people that much. Maybe because we've been in this pandemic for so long, it's changed things, but it's definitely this program, it's taught me that I can connect to people, especially teenagers really well. Especially since maturity levels are somewhere around theirs too." and "I guess I learned that I absolutely loved talking to other people collaborating with others and the mentoring aspect, you know. I just love being able to be surrounded by people – beforehand I wasn't."<br>• **Connection to staff:** "The [program] staff has just been incredible. They've been really, really nice to us and honestly, I didn't think I'd be as close to them because I felt like at the beginning I thought I would just be working alongside them. But now, it feels easier to talk with them and feels more personal. I know them more and they're just really awesome people."<br>• **Connecting with scholars around teaching**: "I like the conversations that we have when they want to be engaged. They are very interesting to talk to and when they want to be vocal about what they don't know. I enjoy teaching them what I do know and transferring that knowledge."<br>• **Connecting despite the virtual setting**: "Since this was in a virtual setting, I wasn't really expecting there to be like, a lot more engaging, I guess, with scholars. But I'm glad I was proved wrong on that because we had so many different opportunities to get to know each of our scholars and I'm glad we did."

Peer mentors' roles and advice for future mentors

Table B7. Mentorship roles and advice to future mentors of cancer research training programs.

Code	Definition	Example Quotes
**Mentorship Roles**	Peer mentors describe roles of peer mentors when working with scholars in the program	• **Guiding scholars in learning research**: "We got to facilitate meetings with our scholars at the end of the day so deeply reflect on what we've been doing. The program has been really helpful for us because we had to facilitate the conversation, make sure we kept it going. We asked them questions or they ask us and having that opportunity during the research rotation, we work with our scholars and [made] sure they were on track, [...] and speaking with their lab's researchers. [I]t was really nice because we were taking the role of a staff member [and] that kind of helped us understand that position [and be] more involved.<br>• **Supporting balance**: "What is mentoring somebody and I think for me, it's like guidance, not just in academics [but] a mix of everything; finding a balance both academic and personal life. Being there for support, giving advice, helping them like they're meant to prepare so they're better off compared to when you were in their position."<br>• **Recognition of challenging times and providing safe spaces**: "It was just really nice to have such a safe place with them and then we were able to talk freely. [Being] flexible and accommodating of [...] personal lives and understanding that [...] everyone has a lot going on."
**Advice for future mentors**	Advice and recommendations to future peer mentors	• **Build trust first**. "I would probably tell [future peer mentors] to be more relaxed and try to connect with the scholars before anything. Earn their trust and try to build some sort of relationship with them instead of just trying to mentor them right away because they're not going to listen if you don't have some sort of relationship with them already. I feel like mentor is kind of a funny word."

Qualifications and experiences helpful for peer mentors

Table B8. Qualifications and experiences of peer mentors that are helpful in cancer research training programs.

Code	Definition	Example Quotes
**College experience with diverse prior experience in research**	Describes diverse backgrounds of peer mentors that support training of high school students, including years in school, prior research experience, being from similar communities, demographics, etc.	• **College experience**: "I think having some years of experience of college education would really help because some of the advice that we had that we were giving them throughout the whole program. We learned through being in college."<br>• **College experience and about to start in research**: "I would be a better peer mentor [now] compared to mentoring when I was just a freshman in college because I've done a lot since then I've had more experiences and I'm older as well. And because I'm also part of the [NIH-funded] BUILD EXITO research program, [I'm familiar with] research ethics and how to conduct research. But I haven't yet performed research in an actual lab. I'm starting next week, so it's exciting if I like where I am now, just like I have and all the other clinical experiences that I've had in the past, and since I've been in college for a few years now, I've been able to help scholars [learn] more about how it's like to be in college, what I did to get it to go to college, [and] support and resources. I feel like I've been able to convey that really well because I've had plenty of experiences since then [high school]."<br>• **Undergraduate with prior research experience**: "I'm in [a BUILD EXITO] research learning community right now and I'm actually working on my own project. So, I think for their community research learning project, I was able to go through with them, like, I am going through it right now [in my own lab] and kind of understand what questions they might have and guide them through what I have done and why. So I think like [identifier redacted] said, just having experience, I'm able to answer more of those questions."<br>• **Recent college graduate with prior research experience:** "I also got to do years of research before so I feel like I experienced a lot and I was able to help them do their research experience as well. Since I graduated, I'm searching long-term jobs and I shared a few things along the way, kind of what it's like after graduation. I think I'm a better mentor in this stage than earlier stages."
**Similar backgrounds as trainees**	Describes similar geographical backgrounds (e.g., communities) and similar experiences (e.g., first generation college, age) that enhance relatability	• **Similar background and community:** "I think that being from a similar background or community has really helped with building relationships with our scholars because they [live in the same area as us]. We're just able to connect better because of a similar education system [and] we are familiar with the area and able to connect in a deeper way compared to someone who was [from different] areas in Oregon."<br>• **Program experience and geographical connection with target audience.** "I agree, being in BUILD [EXITO] and where I am now has helped. I was from the same community as some of the scholars so I was able to relate to them and they were able to ask me really good questions about what I've done in college and stuff like that, so I feel like I was able to relate to them and help them."<br>• **Non-traditional student experience:** "Not being the best student in school here relates to a lot of them in a lot of ways. Also being a non-traditional student. This is my 2nd time at college and I [spent] over a decade in the workforce. I've gotten to work with a lot of different people in a lot of different environments so it made it easier for me to connect with all the scholars and everything."<br>• **Similar experiences**: "Being a 1st generation college student and how that has helped, you connect with the scholars because of questions they had about college or scholarships and I'm able to answer yes."<br>• **Near-peer is important for relatability**: "I think as far as where people are in school, I don't think that matters. I just think the fact that the pyramid choice offer either in college or recent graduates helps because it seems like the scholars relate to that. More so, than if they felt like they were talking to adults."
**Interprofessional peer mentor team**	Describes value of different backgrounds represented among the mentoring team	• "I think that might be hard because we all have such different backgrounds, but we're also kind of heading to the same place like a lot of us want to work in research or healthcare, [...] some type of engineering, but we're all someone who wants to pursue higher education. Maybe that is a good characteristic of peer mentors for this specific program."

Considerations for involving peer mentors in cancer research training programs

Table B9. Considerations and areas for improvement when involving peer mentors in cancer research training programs.

Code	Definition	Example Quotes
**More connection**	Peer mentors describe communication that would help with the mentoring experience	**Earlier connection with trainees:**<br>• "I think maybe having more opportunities [with] the scholars earlier on would have made the program a little less rocky and a little less awkward."<br>**Connection with prior peer mentors**<br>• "I think it might help if we had an opportunity to meet the [peer] mentors they had before. Or, at least be able to pick their brains for information so we have some idea how to interact with the scholars."<br>• Moderator: "Is that something you'd be willing to do for the next cohort of peer mentors? You think it would be useful for them [future peer mentors] would be to meet and chat?" [Notes indicate yes, with agreement in the chat]
**More shared experiences**	Peer mentors describe wanting more time with scholars through shared experiences that could be debriefed	• "I know in the beginning, they [the program] weren't really sure how the peer mentors would really be incorporated into the program and I wish I would have had more time with the scholars in the very beginning because I think we were only attending the debrief at that point instead of being there the whole day, like how we started the 3rd or 4th week."<br>• "I think [a peer mentor] actually did request there to be more because we wanted to get to know the scholars more and we felt like we could help more. But, since the [Immersion] program is new, they weren't really sure how it was going to be organized so we were just kind of going with the flow basically. We were only there for like minutes a day in the beginning and it didn't work out very well. But I feel like the way after got so much better. We got to have more interactions with the scholars, got to be more involved in the program, but I wish that it happened early on."
**Transparency around structure and opportunities**	Includes clarifying program structure and roles in the program, including session participation	• "I remember at the beginning of the program, we had to decide or rank the different projects that we wanted to lead [e.g., photovoice, community research project, scholar social activities], but we didn't really quite know what we were going to get ourselves into. At that time, more help on that would have been appreciated."<br>• "Structure would have been helpful, maybe a little bit more and more interpersonal engagement early on. More involvement in the day-to-day programs from the start."
**Advice for future programs**	Recommendations for improving mentoring experiences	• **Consider group mentoring sessions and a 1:1 assigned lead for each trainee**. "I don't know if anyone else felt this, that certain scholars gravitate towards certain mentors. I felt like I was playing favorites, even though I wasn't really trying to. [S]o maybe having them as my specific mentees might be more beneficial for them and me in certain parts. [It] would have been nice to have assigned each and that you are their primary point of contact. [Having the trainees together in a] group had its benefits. They both had their benefits."<br>• **Using groups first develops connections that permit better matching of 1:1 mentors:** "I remember at the beginning, we did start up in small groups but we hadn't really decided if we were going to have our own assignment. [Smaller] groups would be more personal but at the same time, with the cost of that, we wouldn't get to know the other scholars that are with the other mentors, right? The big group, we have everybody there, but at the cost of the very, closer interpersonal relationships. But ultimately, we felt like just having a bigger group, it's better just because we had all of the scholars together and they all love being with each other and we can also support each other and answer any questions that we can answer right? I'm guessing there are certain scholars that you were more naturally matched with, in terms of interests or backgrounds, or just personalities and maybe if that was preassigned, you wouldn't have known that, or gotten to have those relationships."<br>• **Acknowledging trainee comfort varied across group sizes:** "[H]aving a combination of group activities and smaller group activities might have been helpful." and "[Some scholars] were more shy so maybe some sort of combination of [opportunities between] big group and then breakout rooms or smaller meet and greets kind of thing in the beginning. There are some scholars that I talked to a lot when we're in smaller groups that I would never talk to when we're all together because they're too shy to say anything, but when we're in small groups they won't stop talking. Even though we're virtual, you still see all these little boxes that feel like they're judging you."

Focus group feedback from high school trainees

Themes and quotes that describe peer mentors by scholar trainees are excerpted below, with full findings and contexts reported elsewhere (Marriott et al., 2022).

Introduction scholar feedback

Table B10. Feedback from Introduction trainees in the one week program held in-person (cohort 1) and virtually (cohort 2).

Prompt	Theme	Cohort 1 (In-Person Program	Cohort 2 (Virtual Program)
**"What parts of the program did you like the most?"**	Meeting other scholars/peer mentors<br><br>(Other themes included "Meeting researchers and professionals"; "Exposure to STEM areas/careers"; and "Tours and facilities" (latter theme described for in-person program only)	• Peer mentors were "super nice, funny, easy to relate to."<br>• The other kids in the program got closer this week—talked and bonded.	• "Talking with our mentors was pretty warming. It was almost like talking to another student, they were very open and interested in the program. It was like they were another student; they would ask questions and they'd answer open heartedly and give us honest answers and good advice."<br>• "I live in a super small town so it's hard to find people with same interests so this allowed her to find other people around the same age that are interested in the same thing; and hearing that other people struggle with the same things that I do."

Immersion scholar feedback

Table B11. Feedback from Immersion trainees in the ten week program held virtually.

Prompt	Theme	Example Quotes from Immersion Trainees
**"What parts of the program did you like the most?"**	Peer Mentors<br><br>(Other themes included "Meeting researchers"; "Diversity"; " Public Health shadow"; "Research Rotations"; "Clinical shadow")	• "Peer mentors were great buddies, we'd talk about how our day went/weekend, it was a nice casual talk, but they were closer to our age and gave us great tips. They'd give us office hours to give us advice on our presentations. I hope they stay next year."<br>• "I liked the research rotations as well as the peer mentors we had. It was great. I liked how they interacted with us, and made it fun. They would use large words and great analogies for us to understand."
