# Supplementary material for: Interprofessional Near-Peer Mentoring Teams Enhance Cancer Research Training: Sustainable Approaches for Biomedical Workforce Development of Historically Underrepresented Students
Source: J STEM Outreach. Author manuscript; Available in PMC 2023 May 11. (PMC10174597; doi:10.15695/jstem/v5i2.10)
Supplement: Appendix C [file NIHMS1893019-supplement-Appendix_C.pdf]

Peer Mentors for Workforce Sustainability – Huerta et al.

Appendix C. Follow-Up Data Collection with Peer Mentors

Perspectives Survey

Survey prompts, data definitions and example quotes of observed themes reported by peer mentors in the cancer research training program.

Instrument

Table C1. Survey prompts used to understand follow-up perspectives of peer mentors.

**Follow-up survey prompts**
• When did you work with Knight Scholars Program? (2019 in-person program; 2021 virtual program)<br>• What was your area of interest when you first applied to work with Knight Scholars Program?<br>• Did your area of interest change as a result of working with the Knight Scholars Program?<br>o (Likert Scale: 0=Not at all; 1= A little bit; 2= A lot)<br>o Branching logic if 1 or more: How did your interests change - in what areas or ways?<br>• Did you become more interested in cancer as a result of working with the Knight Scholars Program?<br>• Did you pursue any cancer-related training, internships, or scholarships as a result of participating with the Knight Scholars Program? You do not have to have to have been awarded. What did you pursue, if so?<br>• Did you pursue getting involved with other mentoring activities that help others as a result of participating with the Knight Scholars Program?<br>• Reflecting on your time with the Knight Scholars Program, what was your most significant professional growth?<br>• What would you want NIH or other programs to know about involving peer mentors in their training programs?<br>• Looking at my own career path, I am wanting to..... (whatever you know about your own trajectory)

Data Coding

Area of interest at the time of peer mentor application

Table C2. Description of interests based on the prompt "What was your area of interest when you first applied to work with the Knight Scholars Program?"

Code	Frequency	Definition	Example Quotes
Clinical interests	8/11 (73%)	Specifically describes a clinical related path, such as MD or dermatology	• "Area of interest was healthcare with a goal of pursuing medicine as a career." • "Clinical"; "Clinical Research"
Non-clinical interests	6/11 (55%)	Describes students interested in non-clinical careers (e.g., psychology, teaching, STEM undergraduate research).	• "Psychology" • "Health Management, BIPOC-oriented health disparities, programs to improve retention and the student experience of underrepresented undergraduates."
Population interest	4/11 (36%)	Describes students interested in working with specific populations (e.g., BIPOC health disparities, resource access of communities)	• "My area of interest was being able to help students with the same background as me. I was really interested in learning about data, working with students and also learning about resources that different communities have."
Cancer interest	1/11 (9%)	Cancer is explicitly mentioned as an interest at their self-reported time of application.	• "I was interested in working with the students as I have an interest in teaching and I wanted to have experience before I committed to pursuing a teaching career. I was also interested in learning more about cancer research."

Evolution of peer mentor interests over time

Table C3. Description of how interests changed based on the prompt "How did your interests change - in what areas or ways?"

Code	Frequency	Definition	Example Quotes
Mentoring for legacy	6/9 (67%)	Describes an interest in pursuing future mentorship opportunities and or implementing teaching into a profession. Context often described as wanting to improve training of future scholars.	• "Working with the youth reminded me of how scary it is to be a first-generation and underrepresented high school student trying to figure out whether college or STEM was meant for me. Hearing their stories, lived experiences, and struggles made me realize that we can help students individually but what needs to change is the system and approach. I originally wanted to become a doctor (MD) but this entire experience reminded me of the type of legacy I want to leave behind, which is to help communities and change institutionalized systems to make the process more equitable, experience more endurable, and process/ funding sustainable, which is why I'll be pursuing a dual medicine/ political role." • "I would say that my interest slightly changed to wanting a more academic approach to medicine. My time with the Knight Scholars Program introduced me to the impacts and value that mentoring/education has on students. This experience coupled with other educational work at OHSU instilled in me a desire to want to teach alongside pursuing a career in medicine. As a result, my goal is to practice medicine in an academic institution such as OHSU." • "This experience helped me realize that I do enjoy being a mentor and helping/guide projects."
Non-cancer interest change	4/9 (44%)	Describes a specific interest in a field that is not cancer, even if the cancer training experience spurred it. Fields include bioinformatics and politics.	• "My interests changed from wanting to become a teacher or going to medical school to wanting to do research primarily and teach on the side. I also decided that I wanted to achieve a PhD and not a medical degree." • "One of the groups from OHSU that presented to the scholars were from the bioinformatics department. I had definitely considered this as a career option, if I decided not to go to medical school. But, after listening to their presentation, asking them questions, and doing my own research into the field, I decided to take the leap and apply solely to bioinformatics graduate programs." • "Hearing from students about their experiences within the program changed my professional interests. Rather than working on a clinical research project, I would like to work on directly addressing the barriers within STEM that marginalized students face."
Cancer-related interest change	3/9 (33%)	Describes a specific interest in cancer or cancer-related care	• "I became more interested in end-of-life care of cancer patients. This program helped reinforce my career interest in which I want to work with people in a clinical setting." • "I learned more about cancer research and I am now still interested in the dermatology field but specifically in skin cancers." • "It did not change a lot besides wanting to know/learn more about cancer research."

Pursuance of cancer after participating with the Knight Scholars Program

Table C4. Description of how peer mentors pursued cancer opportunities based on the prompt "Did you pursue any cancer-related training, internships, or scholarships as a result of participating with the Knight Scholars Program? You do not have to have to have been awarded. What did you pursue, if so?"

Code	Frequency	Definition	Example Quotes
Did not pursue cancer	6/9 (67%)	Describes peer mentors who have not pursued cancer-related training, internships, or scholarships.	• "I have not pursued any as of yet."<br>• "I did not, I was pretty set on clinical psych at this point"
Cancer-related training impact (Either pursued or intends to pursue)	3/9 (33%)	Describes peer mentors who either have already pursued or intend to pursue cancer-related training or experiences.	• Example quotes below
Pursued cancer-related training	2/9 (22%)	Describes peer mentors who have pursued cancer-related training, internships, or scholarships since the program, regardless of being awarded or programs held.	• "I pursued another job at the Knight Cancer Institute as a lab assistant because I wanted to keep working with the institute."<br>• "I tried to pursue a cancer-oriented internship [and was accepted] but [it] was canceled due to COVID."
Intends to pursue cancer-related training	1/9 (11%)	Describes peer mentors who intend to pursue cancer-related training, internships, or scholarships in the near future.	• "I did not get the chance to pursue any research on cancer this term or year but in the future I plan to."

Pursuance of mentoring after participating with the Knight Scholars Program

Table C5. Description of how peer mentors pursued mentoring based on the prompt "Did you pursue getting involved with other mentoring activities that help others as a result of participating with the Knight Scholars Program?"

Code	Frequency	Definition	Example Quotes
Mentoring Impact (Pursued or intends to pursue)	10/10 (100%)	Describes peer mentors who either have already pursued or intend to pursue mentoring experiences.	Example quotes below
Pursued mentoring activities	7/10 (70%)	Describes peer mentors who already pursued involvement in other mentor activities that help others. Activities include professional positions, mentor programs, academic clubs, etc.	• "Yes, I served as a University Studies Peer Mentor after this experience"<br>• "Yes, having such a great time as a mentor in the Knights Scholars Program inspired me to lead a study group to help others in my classes that were struggling."<br>• "Yes. I became a student worker for the On Track team at OHSU. I participated in mentoring and teaching at a middle school level. I also became a peer mentor for PSU transfer students through the Louis Stokes Alliance for Minority Participation (LSAMP) Program."<br>• "Yes! I enjoyed the experience and became more confident in working with an age group younger than myself, so I did apply for a couple other peer-mentoring positions."<br>• "I am now a GRA and GTA for McNair and BUILD EXITO, which are both like a peer mentor-oriented position"
Intends to pursue mentoring activities	3/10 (30%)	Describes peer mentors who intend to pursue getting involved in other mentor activities that help others.	• "I have not pursued any as of yet as my participation in this program was recent, however, I do intend to participate in some in the future. I did have some mentoring experience coming into the Knight Scholars program."<br>• "I am currently in the process of applying to a mentoring program! Mentoring with the Knight Scholars Program was an eye-opening experience. It is enriching to work with a group of scholars, and I want to do more of it."<br>• "I have not but I might apply to come back summer 2022 as a mentor for the [Knight Scholars Program]."
No interest in pursuing mentoring	0/10 (0%)	Describes peer mentors who have no further interest in pursuing other mentor activities.	None; all quotes described at least intention to pursue mentoring

Professional growth after participating with the Knight Scholars Program

Table C6. Description of peer mentors' growth based on the prompt "Reflecting on your time with the Knight Scholars Program, what was your most significant professional growth?"

Code	Frequency	Definition	Example Quotes
Professional Communication	8/10 (80%)	Describes peer mentors becoming more comfortable and confident in their professional communication, including asking & answering questions, advising trainees, and facilitating conversations. Often described in the context of taking on leadership skills, teaching activities, and accountability	• "My most significant growth I think is my ability to convey thoughts and information to others in a digestible fashion. My ability to teach has improved."<br>• "Upon reflection, I realize that my time at the Knight Scholars Program has helped develop my communication skills. This comes in the form of being able to facilitate conversations, answer questions, and advising."<br>• "Speaking in front of a large group setting helped me grow as a professional since I have had little experience with this due to the pandemic."<br>• "Communication. I've always considered communication to be one of my strongest assets, but when I learned about several communication styles used by individuals and groups based on their experience and environment, I realized it was both incorrect and startling. That worked really well for me."<br>• "Practicing my active listening skills, especially around sensitive personal topics, was significant to me."
Mentorship	4/10 (40%)	Describes peer mentors who report growth around helping others. Includes appreciation and continuation of mentorship.	• "The most significant thing to my personal growth was being able to help others with my knowledge/experiences."<br>• "Gaining experience mentoring a different age group was very beneficial to my training."<br>• "Learning about the balance between professionalism and genuine human connection by being a kind, relatable and resourceful mentor. I found a way to communicate with a group that is pretty different than myself by finding common interests and sharing my experiences, to hopefully benefit their future."
Networking	2/10 (20%)	Describes improved networking for career growth. Often overlapped with "professional communication" code, but differed in its explicit mention of networking with professionals	• "For me, it was improving my networking skills. I had the opportunity to meet so many professionals over the summer, which helped me expand my network. It was also beneficial because talking with professionals helped ease the anxiousness that I experienced when speaking with experts in their fields of work. This growth became noticeably apparent during the program's final weeks when I had to staff shadowing sessions. I felt confident in my ability to communicate with professionals vocally and via email."<br>• "Being open to new things has allowed me to reach out to people in different fields."
Leadership	2/10 (20%)	Describes growth around leadership skills	• "I learned more about my own strengths and weaknesses as a leader."<br>• "My most significant professional growth was in taking a leadership role in the research shadow days. I think it really taught me how to keep myself and others accountable and deal with unforeseen circumstances."
Openness to careers	1/10 (10%)	Describes peer mentors who became open to careers that differed from when they first applied to the program.	• "Going through all the presentation sessions with the scholars, I realized that I needed to have an open mind when considering career paths. A year ago, I never once considered anything outside of medicine-- I blame this on not being open minded to all the possibilities. Being open to new things has allowed me to reach out to people in different fields, do informational interviews, and eventually expand on my skill set."

Advice to NIH and other programs for involving peer mentors in training programs

Table C7. Description of what peer mentors want others to know based on the prompt "What would you want NIH or other programs to know about involving peer mentors in their training programs?"

Code	Frequency	Definition	Example Quotes
Relatability	8/11 (73%)	Describes peer mentors ability to be relatable and provide valuable insight to trainees. Examples include long-term academic support, similar/recent experiences, offering comfort, etc.	• "Involving peer mentors enhances the experience of students by providing a long-term academic resource. Peer mentors afford students an opportunity to navigate and grow in an often unfamiliar pathway. This becomes paramount for underrepresented students in less pursued academic pathways such as STEM. In addition, as a peer-mentor, I have benefited from mentor-student relationships. I have been able to improve my communication skills, pedagogical knowledge, and cultural competence."<br>• "Peer mentors are a great asset to have in training programs because a lot of times students can form a closer relationship with peer mentors than other staff and confide in them for help."<br>• "As a former mentee, I felt intimidated and shy speaking my mind to the people leading the program but I was always open to my peer mentor. She was close to my age and recently went through what I was going through and she always had the right resources for me. She was also a voice for me when I didn't want to speak up and called me out when I needed it most."<br>• "Involving peer mentors allows scholars to ask questions not only about the [program] but also about their academic journey. Most of the students mentioned feeling comfortable with peer mentors because they had someone to guide them in their projects and ask questions to."<br>• "The benefits of having a mentor that is closer to one's own level is immense. It is easier to have someone that has just went through a similar experience as a guide than someone that went through it too long ago to properly remember what it felt like."<br>• "It's essential! I think having peer mentors is a major asset because we're closer in age to what [they're] experiencing in life, and we act as a bridge between the high school and career years, as we're just leaving one and entering the other. Some kids their age also gravitate to younger adults, and feel more confident opening up so that's also a benefit."<br>• "Inclusivity brings a lot more than what we anticipate. I think it's great to continue this and to support any other program by having peer mentors."<br>• "Peer mentors are absolutely essential to any program that hopes to prepare a generation of professionals for their work. Small groups (with some opportunities for even smaller sub-groups or even individual mentoring) and open discussions are important. Peer mentors allow younger students to forget the pressures of professionalism long enough to ask the most pressing questions."
Liaison between trainees, program, and partners	3/11 (27%)	Describes the benefits of peer mentors acting as liaison across stakeholders, including between trainees, program staff, and scientist/community partners to improve program experiences and outcomes.	• "Involving peer mentors in programs such as the Knight Scholars Program can be extremely helpful. Mentors act as liaisons between students and faculty, which could help improve the program experience/outcomes. Mentors could enhance leadership, research, and teamwork skills in these programs, further preparing them as they work on their career paths."<br>• "Peer mentors are extremely valuable--they can offer insight that program leaders aren't even aware that they need, I saw that happen a couple times this summer."<br>• "Involving peer mentors has the ability to benefit the experience of both the students and the mentors, and they can be an effective bridge between faculty/ professors and students."
Reassurance of sense of belonging	2/11 (18%)	Describes peer mentoring as a source of reassurance and fostering sense of belonging among trainees.	• "We can provide all these students with internship opportunities and exposure to the medical field but the most memorable experience students will recall is the friends/ mentors they meet, especially those who successfully help students realize that they can see themselves in specific STEM-oriented careers."<br>• "Integrating peer mentors into training programs could reassure them that they belong in the sciences due to their roles as they work through the programs."

Career trajectories of peer mentors in the Knight Scholars Program

Table C8. Description of peer mentors' intended career trajectories based on the prompt "Looking at my own career path, I am wanting to..... (whatever you know about your own trajectory)."

Code	Frequency	Definition	Examples and Quotes
Biomedical Sciences	11/11 (100%)	Describes pursuance of biomedical sciences by NIH/NSF Data Book definitions.*	• Includes biomedical, behavioral and social, and clinical sciences classifications as defined in NIH Data Book tables (2019).
Mentoring	8/11 (73%)	Describes an interest in ongoing mentorship or education of others, including future scholars, trainees, and community members. Includes volunteering and teaching roles.	• "Looking at my own career path I want to pursue an academic research position where I may also teach/guide students."<br>• "To be an educator and mentor in my respective career."
Research	7/11 (64%)	Describes an explicit pursuit or intended pursuit of biomedical research as a career. Often described in an academic setting.	• "Looking at my own career path, I am wanting to expand my experience in the field by volunteering and conducting more research."<br>• "My goal is to be a professor at a research university in the future."<br>• "As I work towards that goal [of becoming a physician], I hope to become involved in similar programs like the Knight Scholars Program to earn more research experience."
Inequities	3/11 (27%)	Describes interest in addressing systemic inequities, including work with underrepresented populations.	• "I want to create more programs such as the Knight Scholars, On Track, etc. for not only a selected few but for every HS student who is interested in learning more about STEM. I wanted to get rid of this competitive model and move towards a system that will help anyone regardless of their capabilities to help them become whatever they want to be."<br>• "I would like to continue mentoring students pursuing STEM-related careers with an emphasis toward underrepresented students."
Graduate school	2/11 (18%)	Describes students pursuing or intending to pursue graduate school. Fields includes PhD and clinical psychology programs.	• "I am wanting to go to grad school for bioinformatics. Possibly get my PhD in bioinformatics, specializing in machine learning. I am very excited about these plans!"<br>• "I am currently pursuing my doctorate degree in Clinical Psychology."
Physician	2/11 (18%)	Describes students pursuing or intending to pursue medical education with the primary goal of training as a physician (any degree).	• "Become a physician."<br>• "Become a Doctor of Naturopathic Medicine and pursue a degree in Integrative Medicine Research to continue my research career as a physician."
Cancer	1/11 (9%)	Explicitly describes cancer in their future career plans.	• "I want to continue doing research after I am done with my undergraduate program. I also look forward to working with young children and teens. I hope I also get more into research programs based on cancer."

*NIH Databook (2019, August). Classification of the NSF-NIH survey of graduate students and postdoctorates in science and engineering (GSS) fields. Accessed January 29, 2022 from https://report.nih.gov/nihdatabook/page/field-classification

Follow-Up Demographics Survey

Survey prompts, data definitions and example quotes of observed themes reported by peer mentors in the cancer research training program.

Instrument

Introduction: Hello peer mentors! The questions below ask about your demographics - no names are included. You may skip any question that you don't feel comfortable answering. Ultimately, these questions will be compiled for the paper to understand the demographics of our peer mentors and how many qualify as underrepresented in STEM according to NIH definitions (National Institutes of Health, 2019; https://grants.nih.gov/grants/guide/notice-files/NOT-OD-20-031.html)

Age: What is your age? (open field)

Gender: What is your gender? (open field)

Pronouns: What are your preferred pronouns? (open field)

RaceEthnicity: Which of the following describes your racial or ethnic identity? Please select all that apply

- Native Hawaiian or Pacific Islander
- American Indian or Alaskan Native
- Black or African American
- Hispanic or Latino/a/x
- White
- Middle Eastern/North African
- Asian

[Coding note: Middle Eastern/North African phrasing is described in McGee (2020) and permits upcoding to White according to the US Census (2021): "White – A person having origins in any of the original peoples of Europe, the Middle East, or North Africa.", yet still permits data disaggregation. Full REALD categories beyond those above are recommended (START, 2021)]

Disability: Do you identify as having a disability or a physical, mental, or emotional condition that may influence your participation in school or other activities? This could be a functional limitation or something for which you have sought accommodations at your school's disability resource center. You do not need to have officially sought or secured accommodations.

- No
- Yes
- Prefer not to answer

LGBTQ: Do you identify as LGBTQIA+?

- No
- Yes
- Prefer not to answer

Language_binary: Do you speak any languages other than English?

- No
- Yes
- Prefer not to answer

Display This Question If Do you speak any languages other than English? = Yes
Language_which What language(s) do you speak? (open prompt)

FGCS: Are you a first generation college student? (i.e., the first person in your family to attend college and receive a bachelor's degree. Older siblings going to college does not disqualify you as a first generation college student.)

- No
- Yes
- Don't know
- Prefer not to answer

FGCS_PG1: Parent/Guardian 1 educational level

- Less than high school degree
- High School diploma or GED (General Education Development) equivalent
- Some college (no degree)
- Associate's degree (AA, AAS, AAOT, etc.)
- Bachelor's Degree (BA, BS)
- Some graduate training
- Master's Degree (MA, MS, MAT, etc.)
- Doctoral Degree (MD, PhD, DDT, DMD, PharmD)

FGCS_PG2: Parent/Guardian 2 educational level

- Less than high school degree
- High School diploma or GED (General Education Development) equivalent
- Some college (no degree)
- Associate's degree (AA, AAS, AAOT, etc.)
- Bachelor's Degree (BA, BS)
- Some graduate training
- Master's Degree (MA, MS, MAT, etc.)
- Doctoral Degree (MD, PhD, DDT, DMD, PharmD)

Rural: Did you grow up in a rural area? (U.S. rural area eligibility: https://data.hrsa.gov/tools/rural-health)

- No
- Yes
- Don't know
- Prefer not to say

HPSA: Did you grow up in a medically underserved area? (Centers for Medicare and Medicaid Services-designated Low-Income and Health Professional Shortage Areas (qualifying zipcodes are included in the file)

- No
- Yes
- Don't know
- Prefer not to say

Zipcode: What was your zip code growing up? (open field)

LowSES: Did you grow up with a low socioeconomic background?

- No
- Yes
- Don't know
- Prefer not to say

FRL: Have you ever been eligible for the Federal Free and Reduced Lunch Program for two or more years?

- No
- Yes
- Don't know
- Prefer not to say

WIC: Have you ever received support from the Special Supplemental Nutrition Program (SNAP) for Women, Infants, and Children (WIC) as a parent or child?

- No
- Yes
- Don't know
- Prefer not to say

Pell: Have you ever been eligible for federal Pell grants? (Definition: https://www2.ed.gov/programs/fpg/eligibility.html)

- No
- Yes
- Don't know
- Prefer not to say

Foster: Have you ever been in the foster care system as an infant/child/adolescent, as defined by the Administration for Children and Families

- No
- Yes
- Don't know
- Prefer not to say

Houselessness: Have you ever experienced houselessness/housing instability, as defined by the McKinney-Vento Homeless Assistance Act (Definition: https://nche.ed.gov/mckinney-vento)

- No
- Yes
- Don't know
- Prefer not to say

Other: We realize we may have not captured everything about your demographics. If you would like to say more about communities you represent, please feel free to share.* (open field)

[*Phrasing from Dr. Mollie Marr to be inclusive of demographic and identity categories not represented (F30MH118762).]
