# Supplementary material for: Interprofessional Near-Peer Mentoring Teams Enhance Cancer Research Training: Sustainable Approaches for Biomedical Workforce Development of Historically Underrepresented Students
Source: J STEM Outreach. Author manuscript; Available in PMC 2023 May 11. (PMC10174597; doi:10.15695/jstem/v5i2.10)
Supplement: Appendix D [file NIHMS1893019-supplement-Appendix_D.pdf]

Peer Mentors for Workforce Sustainability – Huerta et al.

Appendix D. Post-Program Stakeholder Reflections

Peer mentor reflections

Jamboard was used to collect peer mentor feedback anonymously during a group meeting (Table D1).

Table D1. Peer mentors' reflections for supporting future involvement in cancer research training programs.

Code	Definition	Example Quotes
Trainee support	Describes comfort of scholars when working with peer mentors. Includes extending reach beyond staff.	• "Scholars have a different comfort level with peer mentors than they do with [KSP staff, e.g. faculty and liaisons]. We think [scholars] might be more comfortable coming to us for certain things rather than the other staff!"<br>• "There were also office hours for extra support for students."
Peer mentor roles	Describes roles and activities done by peer mentors for extending the training of high school students in cancer research (e.g., scheduling, scholar-facing training website, community research project, evaluations and data collection, photovoice, community work).	• "We had many different roles. We all met as a group with the scholars but some peer mentors had special projects that they were involved in." [One] worked with scheduling, scholar training website, and the community research project. [Another] worked with the community research project, etc. [A third] worked with evaluations and data collection, [a fourth] worked with photovoice. And [the fifth peer mentor] worked with the community."<br>• "Supporting students by giving them guidance and direction for different assignments: lit review, presentations, citing sources, etc."
Areas for growth	Peer mentors described approaches for improving the program's sessions and were interested in leading efforts. Included structure and transparency around project leadership and more time with trainees in research experiences.	**Leadership around how program sessions are developed**<br>• "Allow peer mentors to help create sessions with the scholars. We think this is a great leadership opportunity."<br>• "Being able to work in small groups so all the scholars get to talk. In the debrief with the big groups, some scholars wouldn't talk at all. It's possible that smaller groups occasionally could help them be more engaged."<br>**Structure around projects**:<br>• "Peer mentors should decide what project(s) they want to lead a[t] week #1 or before the program begins. That way we have more time to prepare/get in the mindset of leading that project."<br>• "Having a more in depth description of what the projects the scholars will be doing so we're prepared to support them more fully."<br>• "Having a structured weekly schedule for peer mentors that includes their projects."<br>**More time with scholars in research experiences**<br>• "More hands-on facilitating of shadowing/research rotations. Basically fill in the role that the liaisons were doing."<br>• "In person learning - we would be able to do more activities with scholars and mentors for more engagement."

Staff reflections

Jamboard was used to collect feedback anonymously from Knight Scholars Program staff during a group meeting toward the end of the summer 2021 programs (Table D2).

Table D2. Staff feedback and considerations for involving peer mentors in the Knight Scholar Program.

Code	Definition	Example
Staffing challenges that could be addressed with peer mentors	Staff describe considerations around working with minors in rural training sites and how peer mentor roles could be envisioned to support scholar training	• "In person placement/virtual meeting[s and shadowing] with community partners" [can benefit from participation by peer mentors]. Our program recognized limited staff hours for supervising shadowing experiences, which may be supported by including peer mentors, who also cited wanting to share research experiences with trainees. "I'd argue [staff] liaisons being there all day is too much. We don't have that much time/FTE allocated [in the grant budget]. So what should that look like to be more reasonable?"<br>• Longer-term engagement of peer mentors was envisioned to support better staff coverage across staff schedules and other work responsibilities. As staff were part-time liaisons, there was a "lot of info and hard to know where it was on any given day; hard to keep up". It was envisioned that peer mentors could support the day-to-day experiences and work as a team with regional liaisons to facilitate networking and trouble-shoot emergent issues: "I would recommend us holding some morning and afternoon slots of time to respond if things come up."<br>• Potential solution of involving peer mentors in leadership positions with training programs models after existing efforts (e.g., "[Howard Hughes Medical Institute] model - pairing [high school] and [undergraduates] together in labs for research experiences. [M]entors adored it; [supported] multiple levels of mentorship and leadership."<br>• Mentoring teams were envisioned to support synergy across NIH training initiatives, not competition: "I think we'd want to include some of the sophomores or the alums [because juniors and seniors in BUILD] have summer research hours and are busy." Our program, including multiple mentors on a single team allowed them to share responsibilities and balance their schedules to accommodate their research, academic, and personal lives (including self-care or leave in cases of unforeseen sickness or circumstances).
Logistics around hiring peer mentors	Considerations around human resources for including peer mentors when working with youth in cancer research training programs	• "Time requirements" for supervising high school trainees in a ten week training program can be substantial. There was considerable discussion about how to pay peer mentors for their time. Human resources "classification of [peer mentors] is important. In 2019, they were 'experiential'; in 2021 they were classified as 'student workers'". Considerations around "participant" and "worker" are important [based on program duration, level of access needed, and hiring requirements].<br>• The human resource components are time-consuming, both in staff effort and project management needed to oversee compliance paperwork of peer mentors and students. Having a "program manager point person is great - makes OVV [Office of Visitors and Volunteers, which supports university onboarding] things easier since liaison doesn't need to know everything related to processes."<br>• Many peer mentors knew they wanted to continue work with the program. Early recruitment of peer mentors can support onboarding before the spring rush of other training programs seeking approval: [Starting hiring paperwork early is important], so "need to add VLOC submission [university approval of peer mentors] onto [scheduling documents]". Also supports greater collaborative planning efforts with peer mentors.
